# Clinical efficacy of empiric antibiotic therapy in Chinese patients with cirrhosis and bacterial infections: A multicenter study

**DOI:** 10.1371/journal.pone.0357491

**Published:** 2026-09-15

**Authors:** Liqin Sheng, Xiuding Zhang, Haoda Weng, Qinzhi Deng, Min Deng, Xuwei Wu, Zuxiong Huang, Shourong Liu, Rui Wu, Chunlian Ma, Yao Xu, Jianfeng Zhong, Jie Yang, Yinxia Wu, Huajiang Shen, Feng Ding, Fang Wang, Xuezhen Zhai, Chunxian Peng, Haotang Ren, Jie Jin, Xiangfei Xu, Xiaofei Li, Xiaoting Ye, Guoqing Qian, Shuilin Sun, Xuebing Yao, Haifeng Miao, Qianggu Xiao, Shaoheng Ye, Qing Zhang, Xinyi Xu, Xianbin Xu, Yue Yu, Huilan Tu, Xinrong Zhang, Rui Huang, Qiao Yang, Jifang Sheng, Yu Shi, Xia Yu

**Affiliations:** 1 Haiyan People’s Hospital, Jiaxing, Zhejiang, China; 2 State Key Laboratory for Diagnosis and Treatment of Infectious Diseases, National Clinical Research Center for Infectious Diseases, The First Affiliated Hospital, School of Medicine, Zhejiang University, Hangzhou, Zhejiang, China; 3 Hangzhou First People's Hospital Tonglu Hospital, Hangzhou, China; 4 Department of Infectious Diseases, Ningbo No. 2 Hospital, Ningbo, Zhejiang, China; 5 Department of Infectious Diseases, Affiliated Hospital of Jiaxing University/The First Hospital of Jiaxing, Jiaxing, Zhejiang, China; 6 Mengchao Hepatobiliary Hospital of Fujian Medical University, Fuzhou, Fujian, China; 7 Department of Infectious Diseases, Hangzhou Xixi Hospital, Hangzhou Sixth People's Hospital, Hangzhou Xixi Hospital Affiliated to Zhejiang Chinese Medical University, Hangzhou, Zhejiang, China; 8 Department of Infectious Diseases, The First People's Hospital of Wenling, Taizhou, Zhejiang, China; 9 Linyi People's Hospital, Linyi, Shandong, China; 10 Department of Infectious Diseases, Huzhou Central Hospital, Huzhou, Zhejiang, China; 11 Department of Infectious Diseases, The Fifth Affiliated Hospital of Wenzhou Medical University, Lishui, Zhejiang, China; 12 Department of Infectious Diseases, Affiliated Hospital of Shaoxing University, Shaoxing, Zhejiang, China; 13 Department of Infectious Diseases, Beilun District People's Hospital, Ningbo, Zhejiang, China; 14 The Fifth People's Hospital of Huaian, Huaian, Jiangsu, China; 15 Department of Infectious Diseases, Quzhou People's Hospital, Quzhou, Zhejiang, China; 16 Weifang People's Hospital, Weifang, Shandong, China; 17 Department of Infectious Diseases, Affiliated Hangzhou First People's Hospital, School of Medicine, Westlake University, Hangzhou, Zhejiang, China; 18 Department of Infectious Diseases, Yiwu Central Hospital, Yiwu, Zhejiang, China; 19 Department of Infectious Diseases, The Third Affiliated Hospital of Wenzhou Medical University, Wenzhou, Zhejiang, China; 20 Department of Infectious Diseases, Ningbo First Hospital, Ningbo, Zhejiang, China; 21 The Second Affiliated Hospital of Nanchang University, Nanchang, Jiangxi, China; 22 Department of Infectious Diseases, The First People's Hospital of Xiaoshan District, Hangzhou, Zhejiang, China; 23 Dongguan Ninth People's Hospital, Dongguan Infectious Diseases Hospital, Dongguan, Guangdong, China; 24 Department of Gastroenterology, First Hospital of Yangtze University, Jingzhou, Hubei, China; 25 Gastroenterology and Hepatology, School of Medicine, Stanford University Medical Center, Palo Alto, California, United States of America; 26 Department of Infectious Diseases, Nanjing Drum Tower Hospital, Affiliated Hospital of Medical School, Nanjing University, Nanjing, Jiangsu, China; 27 Department of Infectious Diseases, Sir Run Run Shaw Hospital, School of Medicine, Zhejiang University, Hangzhou, Zhejiang, China; 28 Department of Infectious Diseases, Sir Run Run Shaw Alaer Hospital, School of Medicine, Zhejiang University, Alaer, Xinjiang, China; Charite Universitatsmedizin Berlin, GERMANY

## Abstract

**Background & Aims:**

Bacterial and fungal infections are major drivers of acute decompensation and acute-on-chronic liver failure (ACLF) in cirrhosis, but real-world data on the effectiveness of empiric antibiotic therapy in China are scarce. We evaluated clinical response to empiric antibiotics, identified predictors of non-response, and assessed the impact of response and secondary infection on short-term outcomes in Chinese patients with cirrhosis.

**Approach & Results:**

In this multicenter retrospective cohort from 24 tertiary centers, 1,401 adults with cirrhosis and bacterial or fungal infections were included. Clinical response to first-line empiric therapy occurred in 913 patients (65%). Non-responders had higher MELD/MELD-Na scores, more organ failures and ACLF, and more intense systemic inflammation. In multivariable models, greater ACLF grade 2–3, systemic inflammatory response syndrome, higher neutrophil-to-lymphocyte ratio, C-reactive protein, bilirubin, culture positivity, and lower albumin and mean arterial pressure independently predicted non-response. Non-response was independently associated with 28-day and 90-day mortality (HR 4.20 and 3.16, respectively), with consistent findings in time-dependent and center-clustered sensitivity analyses. Secondary infection developed in 7.0% of patients, was four-fold more frequent in non-responders (13.9% vs 3.3%), and nearly doubled 90-day mortality, whereas microbiological features at secondary infection did not clearly distinguish second-course responders from non-responders. Comparative analyses revealed substantial center-to-center and China-global differences in etiology, infection profile, and multidrug-resistant (MDR) burden.

**Conclusions:**

In Chinese patients with cirrhosis, lack of early clinical response to empiric antibiotics independently predicts short-term mortality and is associated with a higher risk of clinically distinct secondary infection. Our findings support risk-adapted, locally informed empiric strategies.

## Introduction

Infection is one of the most frequent complications of cirrhosis, precipitating acute decompensation and acute-on-chronic liver failure (ACLF) and markedly worsening short-term survival [1,2]. Patients with cirrhosis are particularly susceptible to infection because cirrhosis-associated immune dysfunction is compounded by mucosal barrier disruption and microbial dysbiosis. Impaired goblet-cell and mucus defenses, together with perturbations of the gut-liver and oral-gut-liver axes, promote microbial translocation and systemic inflammation, thereby increasing the risk of infectious complications [3,4]. It has been estimated that bacterial and fungal infection occurred in 19% and 6% patients with decompensated cirrhosis and increase 2 and 4-fold risk of death [5,6].

Empiric antibiotic therapy is the cornerstone of initial management for patients with infections and cirrhosis, as microbiological results are usually delayed and early adequacy of therapy is a key determinant of outcome [7,8]. It has been reported that in cirrhotic patients with spontaneous bacterial peritonitis–associated septic shock, each 1-hour delay in receipt of appropriate antimicrobial therapy was associated with an approximately 1.86-fold higher risk of in-hospital mortality [9]. And the International Club of Ascites (ICA) Global Study showed that a substantial proportion of hospitalized patients with cirrhosis and bacterial or fungal infections fail to achieve clinical response to first-line empiric antibiotics, and that non-response independently predicts poor short-term outcomes [8,10]. However, data specifically addressing the clinical efficacy of empiric antibiotic therapy and the determinants of treatment response in Chinese patients with cirrhosis remain limited. This is particular relevant considering differences in underlying etiologies and in the epidemiologic landscape of infections [11].

To address this gap, we conducted a multicenter study to assess the clinical efficacy of empiric antibiotic therapy in Chinese patients with cirrhosis and bacterial or fungal infections, to identify independent predictors of clinical response, and to assess the impact of response on short-term outcomes.

## Methods

### Study design and patients

In this multicenter retrospective cohort, 1,438 adult patients with cirrhosis and bacterial or fungal infections were consecutively identified among hospital admissions to 24 Chinese centers between January 2018 and September 2024 as described elsewhere [11]. Exclusion criteria are detailed in the supplementary materials. In the study, patients with insufficient clinical information to determine either clinical response or non-response were additionally excluded.

The study was conducted in accordance with the principles of the Declaration of Helsinki and was approved by the Ethics Committee of the First Affiliated Hospital, School of Medicine, Zhejiang University (IIT20230123B-R1). The requirement for written informed consent was waived by the Ethics Committee.

### Data collection

We used a predefined standardized case report form to extract data from the electronic medical record systems of all participating centers. The data were accessed for research purposes in October 2024 to January 2025. See the supplementary materials for detailed information. Patients were followed until death, liver transplantation, or the last available contact within 90 days after admission, whichever came first.

### Definitions

Cirrhosis was diagnosed on the basis of imaging features of a nodular liver, endoscopic evidence of portal hypertension, or clinical signs of hepatic decompensation [12]. Acute-on-chronic liver failure (ACLF) was defined according to the criteria of the EASL–Chronic Liver Failure (CLIF) Consortium [13].

Detailed diagnostic criteria for each type of infection are summarized in the Supplementary material. A second infection was defined as a new nosocomial episode arising after the index infection during the same hospital stay [14]. Persistence, progression, or anatomical extension of the unresolved index infection was not considered a secondary infection. Secondary infections were recorded separately and were not used to determine clinical response to the initial empiric regimen. These criteria were applied uniformly to all infection episodes. Empiric antibiotic therapy was grouped into two main strategies: (1) a “classical” regimen, comprising first- to third-generation cephalosporins, amoxicillin–clavulanic acid, cloxacillin, or fluoroquinolones; and (2) an “MDR-oriented” regimen, which included piperacillin–tazobactam, carbapenems, or ceftazidime/cefepime, with or without the addition of a glycopeptide (or linezolid/daptomycin) [15]. Antibiotic regimens were classified as “adherent” to EASL recommendations when they included at least one agent endorsed in the guidelines (Supplementary Table 1 in S1 File). Non-adherent regimens were further categorized as “weaker” if they provided a narrower spectrum than recommended, or “broader” if they exceeded the recommended spectrum [16]. Escalation of antibiotic therapy was considered present when at least one additional drug was introduced or when treatment was changed to a broader-spectrum agent within the first 5 days. De-escalation was defined as decreasing the number of antibiotics or stepping down to a narrower-spectrum regimen within the same timeframe.

Clinical response was determined by the attending physician using a combination of clinical and laboratory criteria, in accordance with previously published definitions. Clinical and laboratory parameters were reassessed during hospitalization according to routine clinical practice, generally every 1–2 days. Clinically, response was considered present when there was clear improvement in infection- or inflammation-related manifestations (e.g., fever, systemic inflammatory response syndrome) and recovery or stabilization of infection-related organ failures. From a laboratory standpoint, response required a decrease in inflammatory markers, including a fall in white blood cell count and C-reactive protein. In patients with spontaneous bacterial peritonitis, an additional criterion was a reduction of ≥25% in ascitic fluid polymorphonuclear cell count after 48 hours of antibiotic therapy. Clinical non-response was defined primarily by persistent or worsening infection-related manifestations or organ failures and/or lack of improvement in inflammatory parameters [10]. Modification or broadening of antibiotic therapy was considered supportive evidence of treatment failure only when prompted by documented clinical or laboratory non-improvement; changes based solely on microbiological results, drug toxicity, or antimicrobial-stewardship considerations did not determine response status. Progression or extension of the unresolved index infection was considered part of the index episode, whereas a clinically distinct secondary infection was recorded separately and was not used to classify response to the initial regimen. In addition, patients with insufficient clinical information to determine either response or non-response were excluded.

Further details of these definitions are provided in the Supplementary material.

### Statistical analysis

Continuous variables were expressed as median (interquartile range) or mean ± standard deviation, and categorical variables as number (percentage). Between-group comparisons of continuous variables were performed using the Mann-Whitney U test for non-parametric data or Student’s t test for normally distributed data. Categorical variables were compared using Pearson’s chi-square test or Fisher’s exact test, as appropriate.

Risk factors for absence of clinical response to empiric antibiotic therapy were evaluated by univariable and multivariable binary logistic regression, applying a backward stepwise procedure for variable selection. Associations are presented as odds ratios (ORs) with corresponding 95% confidence intervals (CIs).

Multivariate Cox proportional hazards models were used to identify independent factors associated with 28-day and 90-day mortality. To account for variation in response timing, sensitivity analyses were performed using time-dependent Cox models, in which all patients entered as non-responders and were reclassified as responders from the first documented response date onward. Patients who never responded remained in the non-response state until death, liver transplantation, loss to follow-up, or administrative censoring. Separate 28-day and 90-day models used the same covariates as the corresponding primary analyses. In additional sensitivity analyses, the multivariable logistic and Cox models were refitted using robust standard errors clustered by participating center to account for within-center correlation.

All analyses were two-sided, with *p* < 0.05 regarded as statistically significant. Statistical calculations were performed with SPSS 26.0 (IBM Corp., Armonk, NY, USA) and R 4.4.2 (R Foundation for Statistical Computing, Vienna, Austria).

## Results

A total of 1,401 patients with cirrhosis and bacterial or fungal infections were included from 24 participating centers. Most patients were male (63.8%) with a mean age of 62 ± 13 years. Hepatitis B virus infection was the leading cause of cirrhosis (44.8%). The cohort had relatively advanced liver disease, with mean Model for End-Stage Liver Disease (MELD) and MELD-Sodium (MELD-Na) scores of 16.9 ± 8.0 and 19.5 ± 10.5, respectively. Systemic inflammatory response syndrome (SIRS) was present in 766 patients (54.7%). The majority of infections were community acquired (1,211 patients; 86%), whereas 190 patients (14%) had nosocomial infections. The most frequent type was pneumonia (435, 26.7%), followed by spontaneous bacterial peritonitis (SBP) (317,19.4%) and spontaneous bacteremia (233,14.3%). MDR isolates were seen in 258 (35.2%) patients, while 16 (2.2%) patients had XDR isolates. The 28-day liver transplant-free mortality in the study cohort was 10.0%, and the 90-day all-cause mortality was 16.7%.

### Predictors of non-response to initial empiric antibiotic therapy

Clinical response to the empiric antibiotic regimen was observed in 913 patients (65%). Supplementary Table 2 in S1 File shows the baseline characteristics according to clinical response versus non-response. The median time to clinical response with empiric antibiotic therapy was 5 days (IQR 4–7). This interval represented the observed time to fulfillment of the predefined response criteria rather than a prespecified response-assessment window. Compared with patients who responded to empiric antibiotic therapy, those with clinical non-response had higher MELD (20.1 ± 8.8 vs. 15.2 ± 7.0; *p* < 0.001) and MELD-Na scores (23.7 ± 11.6 vs. 17.2 ± 9.1; *p* < 0.001), a higher prevalence of systemic inflammatory response syndrome (69.1% vs. 47%; *p* < 0.001), higher white blood cell (WBC) counts (9.4 ± 7.2 vs. 7.3 ± 5.7 × 10⁹/L; *p* < 0.001), higher neutrophil-to-lymphocyte ratios (12.8 ± 15.6 vs. 7.1 ± 10.7; *p* < 0.001) and higher serum C-reactive protein (CRP) levels at enrolment (61.0 ± 59.9 vs. 42.7 ± 49.8 mg/L; *p* < 0.001). The prevalence of organ failures, including liver (20.1% vs. 6.8%; *p* < 0.001), renal (13.1% vs. 6.5%; *p* < 0.001), circulatory (21.9% vs. 5.6%; *p* < 0.001), respiratory (13.7% vs. 1.6%; *p* < 0.001) and cerebral failure (16.2% vs. 5.3%; *p* < 0.001), was significantly higher in patients without clinical response to empiric antibiotic therapy compared with those who responded. We also found significant differences in ACLF severity, with patients without clinical response showing higher rates of ACLF overall and of ACLF grades 2–3 (*p* < 0.001).

In addition to host factors, the microbiological profile, site of acquisition and type of infection also differed between the two groups (Supplementary Table 2 in S1 File). Patients without clinical response more often had pneumonia (37.5% vs. 29.2%; *p* = 0.046), spontaneous bacteremia (23.4% vs. 13.0%; *p* < 0.001), culture-positive infections (*p* < 0.001), and infections caused by MDR (49.6% vs. 39.2%; *p* < 0.001) or XDR (3.3% vs. 1.1%; *p* < 0.001) organisms. Fungal infections were also significantly more frequent in non-responders than in responders (15.7% vs. 11.1%; *p* < 0.001).

We also examined the choice of empiric antibiotic therapy in patients with and without ACLF at the time of infection diagnosis, as a marker of baseline disease severity. Patients with ACLF were more likely to receive broad-spectrum regimens, including carbapenems, glycopeptides and antifungal agents. As expected, when stratified by empiric regimen, patients with ACLF had a lower proportion receiving an in vitro-effective regimen (12.5% vs. 35.0%; *p* < 0.001) and showed poorer adherence to European Association for the Study of the Liver (EASL) recommendations (*p* < 0.001).

**Fig 1:**
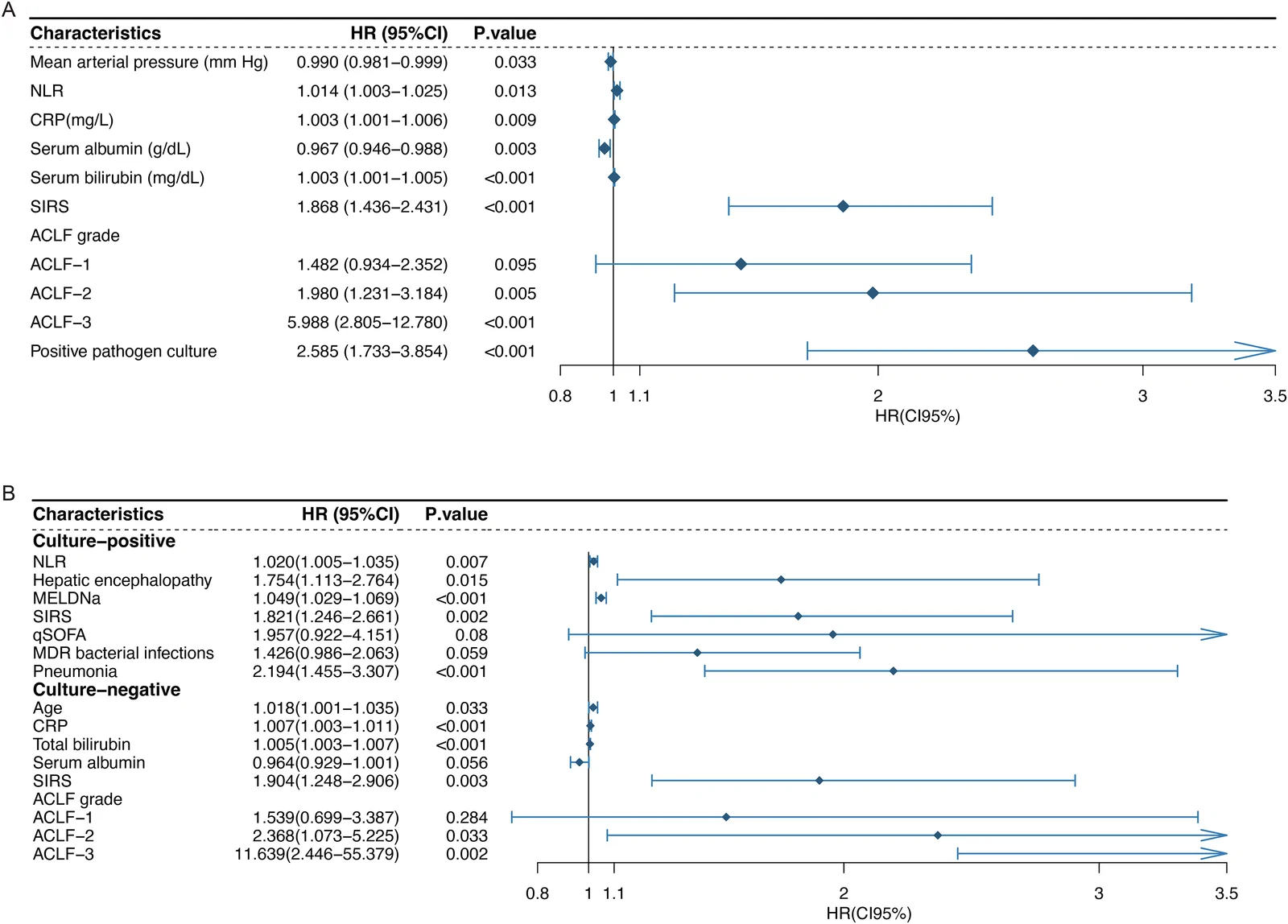
Multivariable logistic regression analyses of risk factors for lack of clinical response to initial empiric antibiotic therapy. (A) Overall cohort. (B) Analyses stratified by culture status, showing separate models for culture-positive and culture-negative infections. Forest plots display adjusted odds ratios (aORs) and 95% confidence intervals (CIs) for non-response to empiric antibiotics after the first infection episode. The vertical line indicates an aOR of 1.0; variables with 95% CIs entirely to the right of this line are significantly associated with lack of clinical effectiveness. Abbreviations: NLR, neutrophil-to-lymphocyte ratio; CRP, C-reactive protein; SIRS, systemic inflammatory response syndrome; ACLF, acute-on-chronic liver failure; qSOFA, quick Sequential Organ Failure Assessment; MDR, multidrug-resistant; MELD-Na, MELD-sodium.

We first performed univariable analyses to screen for variables associated with lack of clinical response to empiric antibiotic therapy (Supplementary Table 3 in S1 File); candidate variables with a p value below a predefined threshold were then entered into a multivariable logistic regression model to identify independent predictors. The severity of systemic inflammation represented by higher neutrophil-to-lymphocyte ratio (NLR) (OR =1.014, 95% CI = 1.003–1.025) and serum C-reactive protein (OR = 1.003, 95% CI = 1.001–1.006) were independent predictors of lack of clinical response to initial antibiotic treatment. The presence of ACLF grade 3 [OR = 5.988, 95% CI = 2.805–12.780] and grade 2 [OR = 1.980, 95% CI = 1.231–3.184] compared to no ACLF (as ref. category OR = 1) were associated with a lack of clinical response to the empiric antibiotic regimen. In addition, positive pathogen culture (OR =2.585, 95% CI = 1.733–3.854), systemic inflammatory response syndrome (OR =1.868, 95% CI = 1.436–2.431), higher serum bilirubin (OR =1.003, 95% CI = 1.001–1.005), lower serum albumin (OR = 0.967, 95% CI = 0.946–0.988) and lower mean arterial pressure (OR = 0.990, 95% CI = 0.981–0.999) were identified as independent factors predicting lack of response to the empiric antibiotic strategies (Fig 1A).

We performed a sensitivity analysis stratified by culture-positive and culture-negative infections to explore factors associated with lack of clinical response (Fig 1B and Supplementary Table 4 in S1 File). Among patients with culture-positive infections, non-responders more frequently had pneumonia and spontaneous bacterial peritonitis (46.4% and 41.2%, respectively), whereas responders more often presented with urinary tract infection (25.0%) or spontaneous bacteremia (36.3%). In culture-negative infections, spontaneous bacterial peritonitis and pneumonia predominated in both responders and non-responders, with very few urinary tract infections or bacteremia. Most patients in each stratum received MDR-covering empiric regimens, and carbapenems were used more often in culture-positive than in culture-negative responders (24.5% vs. 14.1%). In multivariable models, the presence of systemic inflammatory response syndrome (SIRS) was associated with non-response in both culture-positive and culture-negative infections. Among culture-negative infections, younger age, higher C-reactive protein (CRP) and total bilirubin levels, lower serum albumin, and higher ACLF grade were associated with lack of clinical response, whereas in culture-positive infections, pneumonia and hepatic encephalopathy, a higher neutrophil-to-lymphocyte ratio (NLR), and a higher MELD-Na score were associated with non-response.

### Secondary infection, clinical response to antibiotics, and short-term mortality

**Fig 2:**
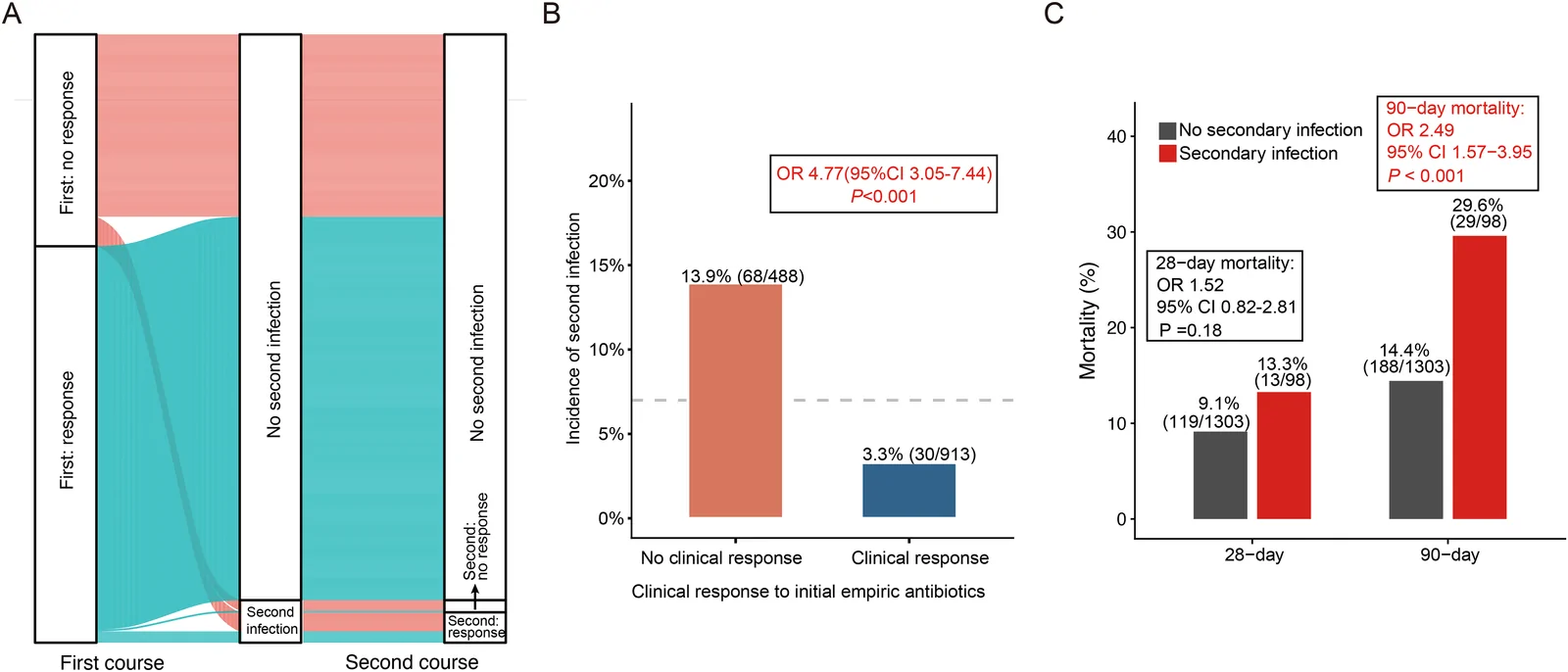
Clinical course and prognostic impact of secondary infection according to response to empiric antibiotic therapy. (A) Patient trajectories according to response to empiric antibiotic therapy and occurrence of second infection. Sankey plot showing the flow of patients from clinical response to the first empiric antibiotic course (left axis), through the development or absence of a second infection (middle axis), to clinical response to second-line antibiotics or no second infection (right axis). Ribbon width is proportional to the number of patients moving between strata, and colors distinguish patients with versus without clinical response to the first empiric regimen. (B) Incidence of a second infectious episode according to clinical response to initial empiric antibiotics. Bars show the incidence of a second infection in patients with and without a clinical response to the initial empiric regimen. Labels above the bars indicate the percentage and the corresponding number of events (n/N). The dashed horizontal line denotes the overall incidence of second infections in the cohort. (C) 28-day and 90-day mortality according to the occurrence of secondary infection. Bars show the 28-day/90-day mortality of patients with and without secondary infection. Labels above the bars indicate the percentage and the corresponding number of events (n/N). ORs and 95% CIs, as well as the corresponding *p* values, were derived from univariable logistic regression models based on the same 2 × 2 contingency tables.

Among 1,401 patients with cirrhosis or ACLF and a first infectious episode, 98 (7.0%) developed a secondary infection during the index hospitalization. By definition, these episodes were distinguished from persistence, progression, or extension of the index infection and were not used to assign response status to the initial empiric regimen. Secondary infection occurred much more frequently in patients who failed to achieve clinical response to the initial empiric antibiotic regimen than in those with response (13.9% [68/488] vs 3.3% [30/913]; odds ratio [OR] 4.77; 95% confidence interval [CI] 3.05–7.44; *p* < 0.001). Although the occurrence of secondary infection had only a modest, non-significant impact on 28-day mortality (13.3% vs 9.1%; OR 1.52, 95% CI 0.82–2.81; *p* = 0.18), it was associated with almost a doubling of 90-day mortality (29.6% vs 14.4%; OR 2.49, 95% CI 1.57–3.95; *p* < 0.001) (Fig 2).

Among the 98 secondary infections, 30 episodes (30.6%) occurred in patients who had responded to the initial empiric regimen and 68 (69.4%) in those without initial response. At the time of secondary infection, the distribution of infection sites, culture positivity rates, and the prevalence of multidrug-resistant (MDR) pathogens were broadly similar between patients with and without clinical response to first-course antibiotics, with the exception that antibiotic-resistant isolates were more frequent when the initial empiric therapy had failed (87.5% vs 36.4%, *p* = 0.003). Overall, 70 of 98 secondary infections (71.4%) achieved clinical response to the subsequent antibiotic course, and patients with versus without second-course response again showed comparable spectra of infection sites, culture positivity, and rates of resistance and MDR organisms (Supplementary Table 5 in S1 File).

Collectively, lack of early clinical response was associated with a higher incidence of a subsequently identified, clinically distinct secondary infection and with higher 90-day mortality. Microbiological characteristics at the onset of secondary infection did not clearly distinguish responders from non-responders to second-line therapy.

### Predictors of 28-day and 90-day mortality

**Fig 3:**
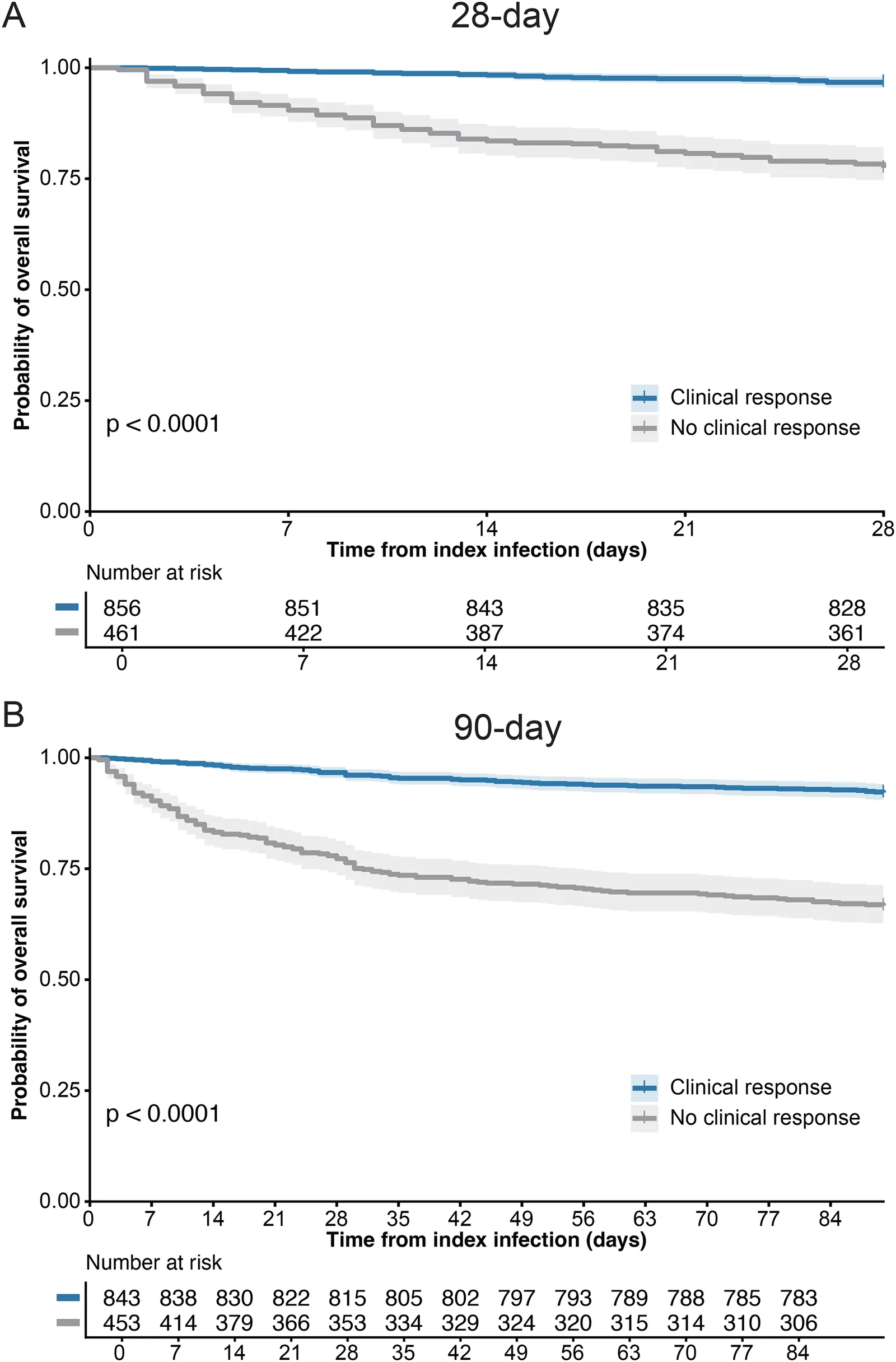
Kaplan–Meier curves for overall survival according to clinical effectiveness of initial empiric antibiotic therapy after the first infection. Kaplan–Meier estimates of (A) 28-day and (B) 90-day overall survival from the first episode of infection, stratified by clinical response to initial empiric antibiotic therapy. Patients who achieved clinical response after empiric antibiotics had a substantially higher probability of survival than those without clinical response across both time horizons (log-rank P < 0.0001 for both comparisons). Time zero was defined as the onset of the index infection, and numbers at risk at each time point are shown below the x-axis.

Baseline characteristics according to survival status are summarized in Supplementary Table 6 and 7 in S1 File. Patients who achieved clinical response to initial empiric antibiotic therapy after the first infection had significantly better 28- and 90-day overall survival than non-responders (log-rank *p* < 0.0001). These findings highlight that early effective empiric treatment of infection is closely associated with short-term prognosis in cirrhotic patients (Fig 3). In multivariable Cox regression (Supplementary Table 8 in S1 File), older age, lower serum albumin, higher MELD-Na, and the presence of hepatic encephalopathy were independently associated with both 28-day and 90-day mortality. In addition, a higher neutrophil-to-lymphocyte ratio (NLR; HR 1.012, 95% CI 1.003–1.022), higher qSOFA score (HR 1.853, 95% CI 1.150–2.985), and septic shock (HR 2.674, 95% CI 1.668–4.287) were associated with 28-day mortality. Male sex (HR 1.538, 95% CI 1.146–2.064) and higher ACLF grade [ACLF-2: HR 1.868 (95% CI 1.170–2.983); ACLF-3: HR 1.982 (95% CI 1.096–3.583)] were associated with 90-day mortality. Clinical response to empiric antibiotic therapy also emerged as an independent predictor of mortality at both time points [28-day: HR 4.200 (95% CI 2.673–6.601); 90-day: HR 3.160 (95% CI 2.308–4.326)].

**Fig 4:**
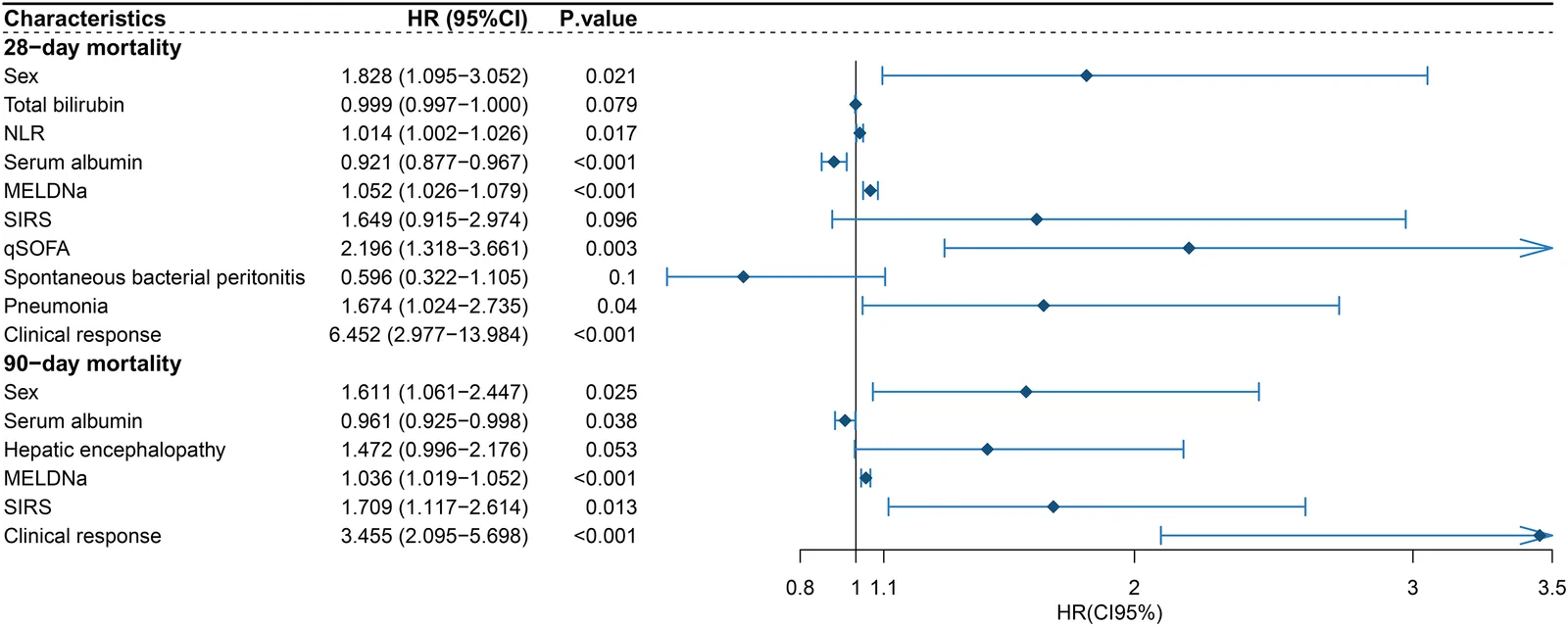
Multivariable Cox regression analysis of predictors of 28-day and 90-day mortality after the index infection in patients with ACLF. Forest plots display adjusted hazard ratios (HRs) and 95% confidence intervals for factors associated with (upper panel) 28-day and (lower panel) 90-day mortality after the first infection episode. The vertical line denotes an HR of 1.0; variables with HRs and 95% CIs entirely to the right of this line indicate increased mortality risk. Abbreviations: NLR, neutrophil-to-lymphocyte ratio; SIRS, systemic inflammatory response syndrome; qSOFA, quick Sequential Organ Failure Assessment; MELD-Na, MELD-sodium.

Of the overall cohort, 277 patients (19.8%) fulfilled criteria for ACLF. Among these, 81 patients (30.7%) died within 28 days and 115 (44.2%) had died by 90 days. We therefore performed a subgroup analysis to identify predictors of 28-day and 90-day mortality in this population (Fig 4). In multivariable Cox regression, male sex, lower serum albumin, higher MELD-Na and lack of clinical response to empiric antibiotic therapy were independently associated with both 28-day and 90-day mortality. Additional independent predictors of 28-day mortality included a higher neutrophil-to-lymphocyte ratio (NLR), higher qSOFA score and the presence of pneumonia, whereas SIRS was also independently associated with 90-day mortality in ACLF patients with infection.

Because clinical response occurred at different times after treatment initiation, we additionally performed sensitivity analyses using clinical response as a time-dependent covariate. In these analyses, patients contributed person-time to the non-response state until the first documented date of clinical response and to the response state thereafter. Patients who did not achieve a documented response, including those who died before achieving clinical response, remained in the non-response state until death or censoring.

After accounting for the timing of clinical response, the absence of clinical response remained independently associated with a higher risk of both 28-day mortality (HR = 5.632; 95%CI = 3.698–8.578; p < 0.001) and 90-day mortality (HR = 2.966; 95%CI = 2.205–3.990; p < 0.001). The direction and statistical significance of these associations were consistent with the primary analyses, indicating that the observed prognostic association was not solely attributable to immortal-time bias.

### Clinical response to empiric antibiotic therapy by geographic region

**Fig 5:**
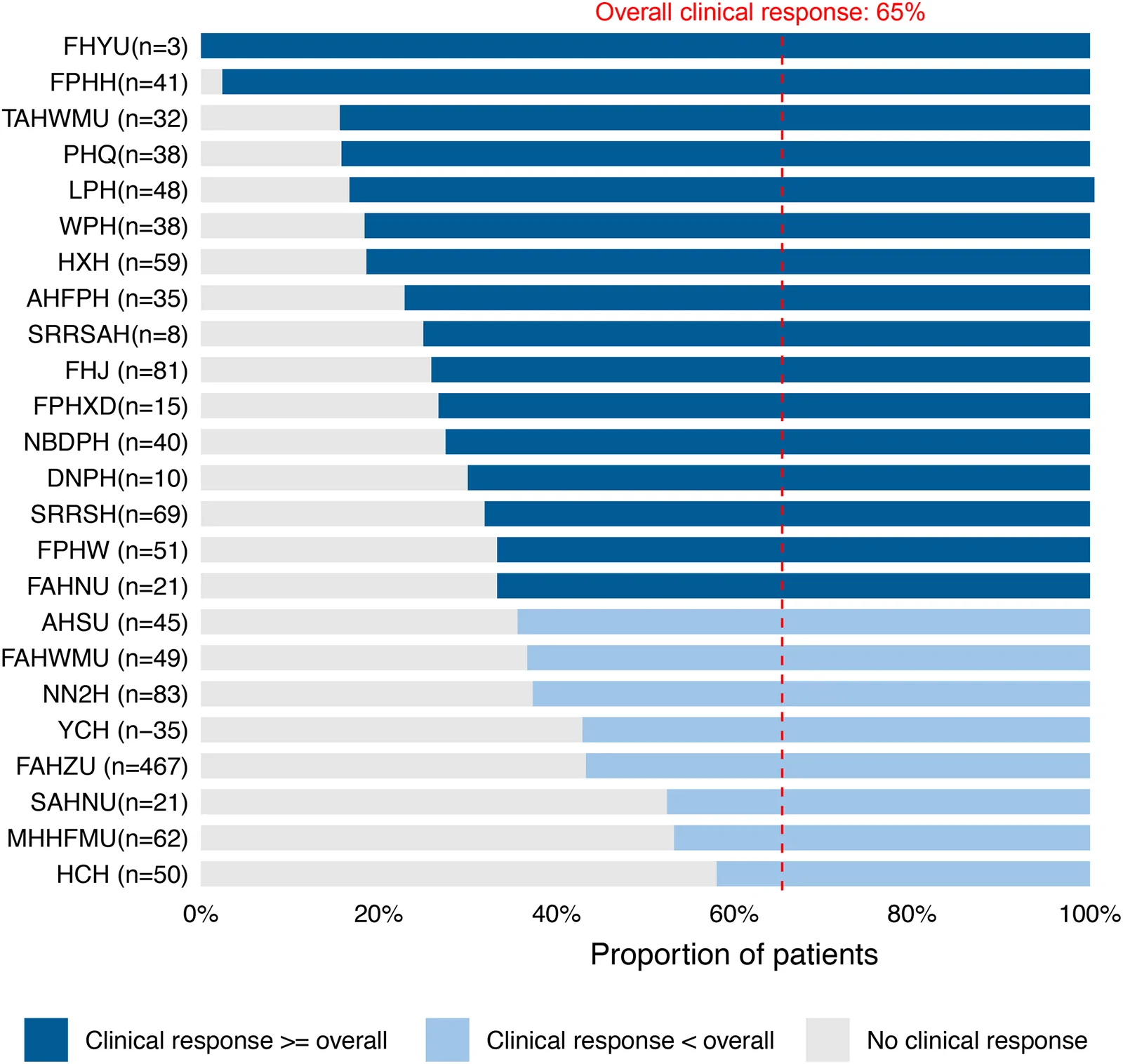
Center-level variation in clinical response to empiric antibiotic therapy. Horizontal stacked bars show, for each participating hospital, the proportion of patients with clinical response (blue) and no clinical response (light grey). Centers are ordered by increasing clinical response rate. The vertical dashed line indicates the overall, sample-size–weighted clinical response rate (65%) in the study cohort. Dark blue segments denote centers with a clinical response rate ≥ the overall rate, whereas light blue segments denote centers with a clinical response rate < the overall rate. Abbreviation: FAHZU, The First Affiliated Hospital, Zhejiang University School of Medicine; NBDPH, Ningbo Beilun District People’s Hospital; NN2H, Ningbo No.2 Hospital; FAHNU, The First Affiliated Hospital of Ningbo University; YCH, Yiwu Central Hospital; TAHWMU, The Third Affiliated Hospital of Wenzhou Medical University; FHJ, The First Hospital of Jiaxing; FAHWMU, The Fifth Affiliated Hospital of Wenzhou Medical University; AHSU, Affiliated Hospital of Shaoxing University; AHFPH, Affiliated Hangzhou First People’s Hospital; FPHW, the First People’s Hospital of Wenling; HXH, Hangzhou Xixi Hospital; HCH, Huzhou Central Hospital; FPHXD, The First People’s Hospital of Xiaoshan District; SRRSH, Sir Run Run Shaw Hospital; PHQ, The People’s Hospital of Quzhou; LPH, Linyi People's Hospital; WPH, Weifang People’s Hospital; SRRSAH, Sir Run Run Shaw Alaer Hospital; DNPH, Dongguan Ninth People’s Hospital; FHYU, First Hospital of Yangtze University; FPHH, The Fifth People’s Hospital of Huaian; MHHFMU, Mengchao Hepatobiliary Hospital of Fujian Medical University; SAHNU, The Second Affiliated Hospital of Nanchang University.

As shown in Fig 5, clinical response to empiric antibiotic therapy also varied substantially across hospitals, with response rates ranging from 42.0% at Huzhou Central Hospital to 100.0% at the First Hospital of Yangtze University. In the largest recruiting center (FAHZU), 56.7% of patients achieved clinical response (265/467), whereas several other centers reported response rates above 80% (e.g., TAHWMU, PHQ, LPH, WPH, HXH), and a few centers had roughly equal or even higher proportions of non-responders (e.g., HCH, MHHFMU, SAHNU). These findings underscore considerable inter-center heterogeneity in clinical outcomes despite broadly similar empiric treatment strategies. Sensitivity analyses using center-clustered robust standard errors were consistent with the primary results. The direction and magnitude of the main associations with clinical non-response remained materially unchanged, and lack of clinical response remained independently associated with 28-day mortality (HR = 3.984; 95%CI = 2.526–6.283; p < 0.001) and 90-day mortality (HR = 3.083; 95%CI = 2.242–4.242; p < 0.001).

**Fig 6:**
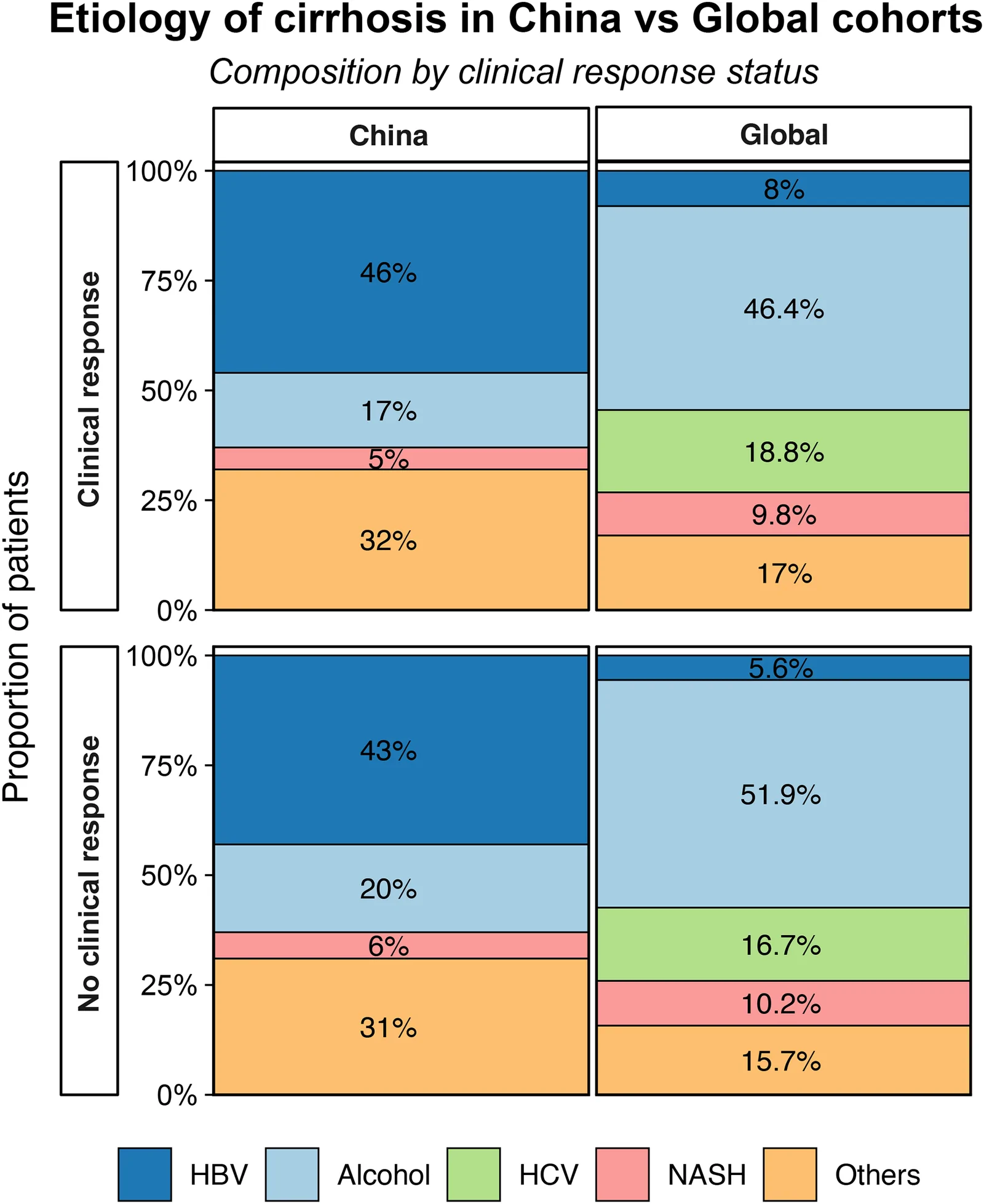
Etiology of cirrhosis in the Chinese and global cohorts, stratified by clinical response. Stacked 100% bar charts display the proportional distribution of underlying causes of cirrhosis in patients with (upper panels) and without (lower panels) clinical response, comparing the Chinese (left) and global (right) cohorts. Each bar represents the relative contribution of hepatitis B virus (HBV), alcohol-related liver disease, hepatitis C virus (HCV), nonalcoholic steatohepatitis (NASH), and other etiologies within each subgroup, with percentage values shown inside the segments. Data are expressed as the proportion of patients within each cohort and response category. Abbreviation: HBV, hepatitis B virus; HCV, hepatitis C virus; NASH, nonalcoholic steatohepatitis.

**Fig 7:**
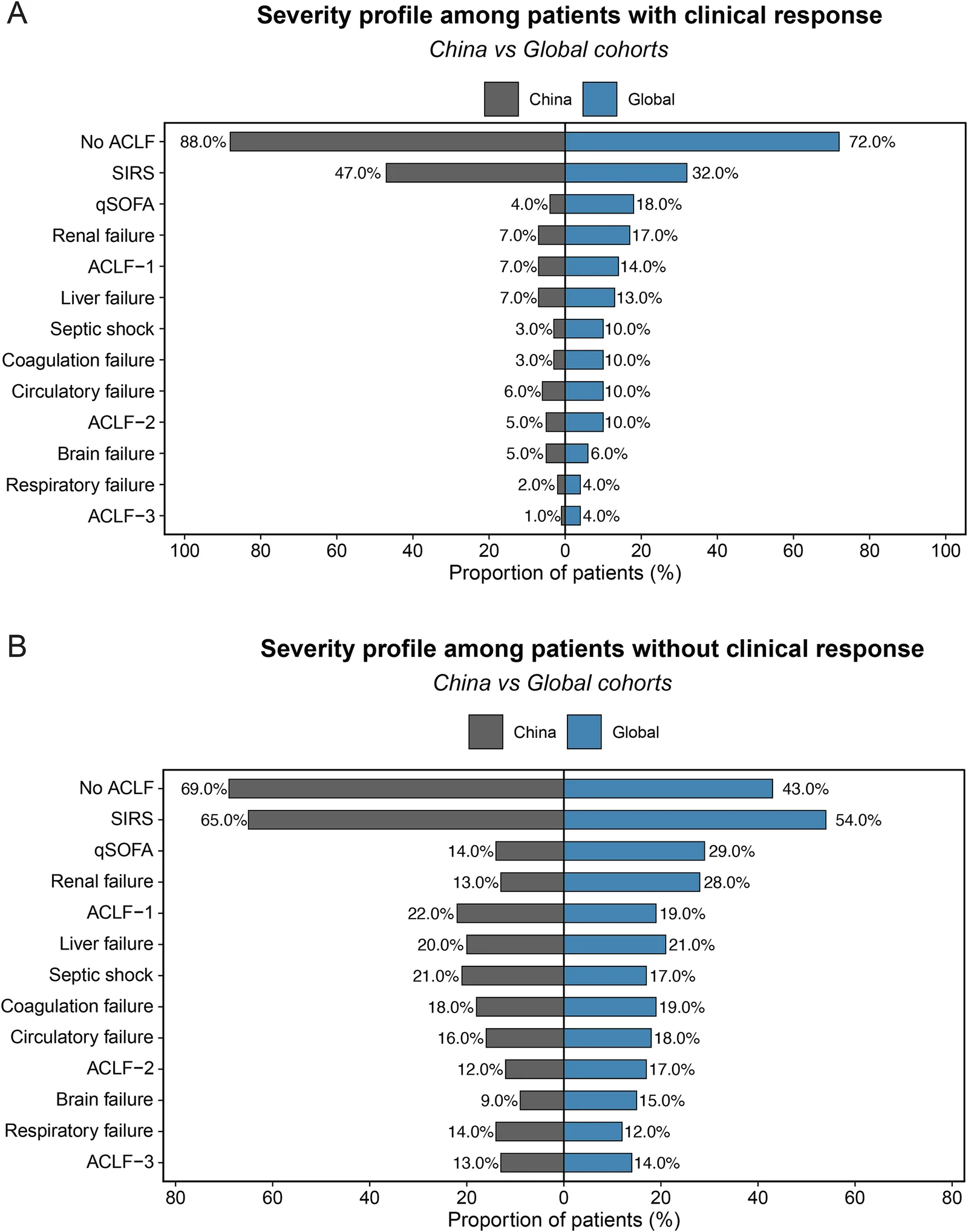
Severity profile among patients without clinical response in the Chinese and global cohorts. Back-to-back horizontal bar charts depict the proportion of patients with each severity-related characteristic among those without clinical response, with bars to the left of the central axis representing the Chinese cohort and bars to the right representing the global cohort. Bar length reflects the percentage of patients in each cohort, with numeric values displayed adjacent to each bar. Abbreviation: ACLF, acute-on-chronic liver failure; SIRS, systemic inflammatory response syndrome; qSOFA, quick Sequential Organ Failure Assessment.

**Fig 8:**
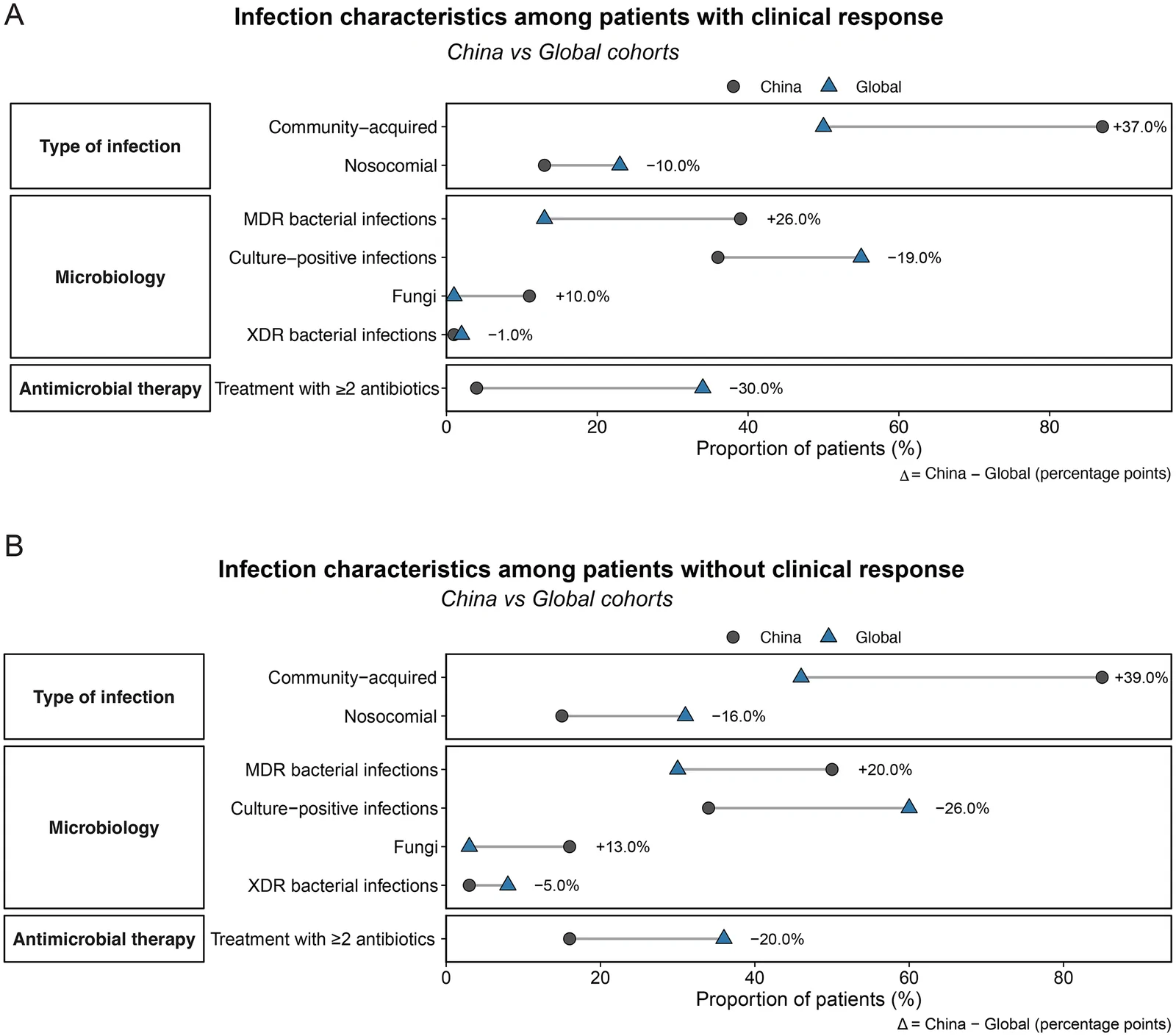
Infection characteristics among patients with clinical response in the Chinese and global cohorts. Dumbbell plots depict the proportion of patients with each infection-related feature among those achieving clinical response (A) and those who did not achieve clinical response (B), comparing China (solid circles) and the global cohort (open triangles). Horizontal lines connect the two cohorts for each variable, with Δ values (percentage points) shown to the right and calculated as China minus Global. Variables are grouped into three domains (type of infection, microbiology, and antimicrobial therapy). Data are expressed as percentages of patients within each cohort. Abbreviation: MDR, multidrug-resistant; XDR, extensively drug-resistant.

Geographic differences were also evident when comparing the Chinese cohort with the global ICA cohort stratified by clinical response (Supplementary Table 9 in S1 File). Chinese patients with clinical response were older but more often had HBV-related cirrhosis and less frequently alcohol- or HCV-related disease than those in the global cohort (Fig 6); they also had lower MELD and MELD-Na scores, fewer organ failures, and a higher proportion of community-acquired rather than nosocomial infections. Among both responders and non-responders, Chinese patients showed a predominance of pneumonia and spontaneous bacteremia and fewer urinary tract infections, together with fewer culture-positive episodes overall but a higher proportion of MDR infections and more frequent fungal infections, and were less often treated with combinations of two or more antibiotics (Figs 7 and 8). Taken together, these data indicate that clinical response to empiric antibiotic therapy is influenced not only by patient- and infection-related factors but also by geographic and institutional context, with substantial variation across centers within China and between the Chinese and global cohorts.

## Discussion

Our multicenter cohort characterizes the real-world effectiveness of empiric antibiotic therapy in hospitalized Chinese patients with cirrhosis and bacterial or fungal infections. Approximately one-third of patients did not achieve a clinical response to first-line empiric treatment, a rate that closely mirrors the ~ 40% non-response reported in the ICA Global Study [10]. This convergence across geographically and etiologically distinct populations underscores that suboptimal early antibiotic efficacy is a consistent and clinically important problem in cirrhosis [17]. Non-response was more likely in patients with more advanced stage, a higher burden of organ failures and acute-on-chronic liver failure (ACLF), and more intense systemic inflammation, and remained independently associated with both 28- and 90-day mortality after adjustment for established prognostic factors. These findings extend prior work showing that infection precipitates acute decompensation and ACLF, and that the adequacy of the initial antimicrobial regimen is a key determinant of survival.

Disease severity and systemic inflammation were major drivers of treatment failure [18]. Non-responders had higher MELD/MELD-Na scores, more extrahepatic organ failures, and a greater prevalence of ACLF—particularly grades 2/3—than responders. In multivariable analysis, ACLF grade 2–3, lower mean arterial pressure, higher bilirubin, lower albumin, and the presence of systemic inflammatory response syndrome (SIRS) independently predicted lack of clinical response, consistent with the concept of cirrhosis-associated immune dysfunction and the central prognostic role of organ failure in decompensated cirrhosis and ACLF [19–21]. These variables are readily available at the bedside and could be incorporated into simple risk-stratification tools to identify patients at high risk of early empiric treatment failure.

Markers of systemic inflammation and sepsis severity also emerged as important correlates of outcome. Non-responders had higher white blood cell counts, neutrophil-to-lymphocyte ratio (NLR), and C-reactive protein (CRP) concentrations; NLR and CRP independently predicted non-response, while NLR, qSOFA and septic shock were associated with 28-day mortality, and qSOFA and SIRS remained relevant in patients with ACLF. These data support NLR as a simple, inexpensive marker of systemic inflammation and short-term prognosis in decompensated cirrhosis and HBV-related ACLF [22,23], and suggest that Sepsis-3–derived tools such as qSOFA can assist bedside risk stratification in infected patients with cirrhosis [24]. Composite profiles combining ACLF grade, hemodynamics, bilirubin, albumin, NLR/CRP and qSOFA/SIRS may help clinicians to identify patients who warrant more intensive monitoring, early hemodynamic support, expanded diagnostic work-up, and a lower threshold for escalation of antimicrobial therapy [1,25].

Beyond host factors, the infection profile and local microbiology were closely associated with treatment response. Pneumonia, spontaneous bacteremia and culture-positive infections were more frequent among non-responders, who also had higher rates of multidrug-resistant (MDR) or extensively drug-resistant (XDR) pathogens and invasive fungal infections. This pattern is in line with global trends toward more severe pneumonia and bloodstream infections and a growing burden of MDR Gram-negative, Gram-positive and fungal pathogens in hospitalized patients with cirrhosis [7,15,26]. In the subgroup of patients who developed a second infectious episode, MDR organisms were significantly more common in non-responders, despite similar overall culture positivity and a comparable distribution of Gram-negative, Gram-positive and fungal isolates [27–29]. Because antibiotic escalation may itself be a consequence of perceived treatment failure, we did not interpret its distribution across response groups as an independent or causal association. The retrospective design also precludes firm conclusions regarding whether microbiological severity caused treatment failure or instead reflected greater underlying disease severity and healthcare exposure.

Secondary infection in cirrhosis is now recognized as a clinically relevant “second hit” on a background of cirrhosis-associated immune dysfunction and a rising global burden of multidrug-resistant (MDR) organisms [8,15]. In our cohort, only 7.0% of patients developed secondary infection during the index hospitalization, but this complication clustered in those without early clinical response to empiric antibiotics, in whom the risk of secondary infection was more than four-fold higher and translated into almost a doubling of 90-day mortality, while exerting little effect on 28-day mortality. This pattern is consistent with the dynamic view of acute decompensation described in the PREDICT study and ACLF guidelines, where failure to resolve the initial precipitating event identifies an unstable trajectory with excess medium-term mortality rather than immediate death [30]. Notably, once a secondary infection had occurred, baseline microbiological features, including site of infection, culture positivity, and MDR prevalence were broadly similar between subgroups and did not clearly distinguish responders from non-responders to second-line therapy, despite a higher frequency of resistant isolates in patients whose first empiric regimen had failed. This observation aligns with large intercontinental studies showing that MDR infections in cirrhosis are common, treatment-refractory, and tightly linked to prior antibiotic exposure and healthcare contact, but that outcomes are also strongly modulated by underlying liver failure and host immune dysfunction [31]. Together, these data support that lack of early clinical response to empiric antibiotics should be regarded as a high-risk state that warrants intensified surveillance for secondary infection, early reassessment and adaptation of therapy, and incorporation into prognostic stratification in line with contemporary guideline recommendations.

A novel aspect of this study is the direct comparison between the Chinese cohort and the ICA Global cohort. Chinese patients were older, predominantly had HBV-related rather than alcohol- or HCV-related cirrhosis, had lower MELD/MELD-Na scores and fewer organ failures, and more often presented with community-acquired infection [32,33]. Despite this apparently lower baseline risk, MDR and fungal infections were more frequent, whereas urinary tract infections and culture-positive episodes were less common, and combinations of two or more antibiotics were used less often. These geographic differences echo prior reports that MDR prevalence, infection spectra, and prescribing patterns vary substantially between regions and even between hospitals, reinforcing the need for locally adapted empiric strategies rather than uniform global algorithms. At the health-system level, regional and institutional differences in cirrhosis etiology, infection acquisition, pathogen ecology and antimicrobial resistance, availability and timing of microbiological diagnostics, prescribing and stewardship practices, source-control capacity, and access to critical care may further modify treatment response and outcomes. These factors may contribute to inter-centre variation beyond differences in case mix and support local surveillance and centre-specific empiric treatment protocols [14,34,35].

The heterogeneity in treatment response may also reflect interactions between host biology and the infectious insult. Disruption of the intestinal barrier and microbial dysbiosis can promote microbial translocation, while cirrhosis-associated immune dysfunction and dysregulated inflammatory responses may impair pathogen clearance and amplify tissue injury. Bidirectional organ-to-organ crosstalk, particularly along the gut-liver axis and between the liver, kidneys, lungs, and systemic circulation, may further propagate inflammation and organ failure, thereby influencing whether infection resolves with initial therapy or progresses despite antimicrobial treatment. Although these mechanisms were not directly assessed in our study, they provide a plausible framework for the observed associations of SIRS, ACLF severity, hemodynamic impairment, and extrahepatic organ failure with non-response and mortality. Such cell-to-cell and organ-to-organ interactions have been characterized particularly in alcohol-associated liver disease and may have broader relevance to advanced cirrhosis [36].

This study has limitations. First, its retrospective design introduces potential residual confounding, and clinical response was defined by treating physicians, although standardized criteria aligned with ICA definitions and supported by laboratory data were applied. Second, antibiotic modification is both a management response to perceived treatment failure and a marker of clinical severity; therefore, comparisons involving escalation were regarded as descriptive and not interpreted causally. Although secondary infection was defined and recorded separately from progression of the index infection, residual reverse causality between evolving response status, subsequent infection, and mortality cannot be completely excluded. Third, microbiologic work-up was not completely uniform across centers, pharmacokinetic data were unavailable, and information on source control was incomplete. Finally, the cohort was derived from tertiary Chinese centers, which may limit generalizability to other settings.

In conclusion, this multicenter Chinese study shows that failure to achieve a clinical response to empiric antibiotics in hospitalized patients with cirrhosis is driven by advanced liver disease, systemic inflammation, high-risk infection phenotypes and MDR pathogens, and is strongly and independently associated with short-term mortality. Together with global data, these findings support risk-adapted, locally informed empiric antibiotic strategies and highlight the need for prospective trials testing tailored, region-specific treatment algorithms.

## Supporting information

S1 File Supplementary appendix. Supplementary Methods, Supplementary Tables 1–9, and Supplementary References.
(DOCX)
