# Correction: Clinical efficacy of empiric antibiotic therapy in Chinese patients with cirrhosis and bacterial infections: A multicenter study

**DOI:** 10.1371/journal.pone.0361389

**Published:** 2026-10-23

**Authors:** Liqin Sheng, Xiuding Zhang, Haoda Weng, Qinzhi Deng, Min Deng, Xuwei Wu, Zuxiong Huang, Shourong Liu, Rui Wu, Chunlian Ma, Yao Xu, Jianfeng Zhong, Jie Yang, Yinxia Wu, Huajiang Shen, Feng Ding, Fang Wang, Xuezhen Zhai, Chunxian Peng, Haotang Ren, Jie Jin, Xiangfei Xu, Xiaofei Li, Xiaoting Ye, Guoqing Qian, Shuilin Sun, Xuebing Yao, Haifeng Miao, Qianggu Xiao, Shaoheng Ye, Qing Zhang, Xinyi Xu, Xianbin Xu, Yue Yu, Huilan Tu, Xinrong Zhang, Rui Huang, Qiao Yang, Jifang Sheng, Yu Shi, Xia Yu

The affiliation for the ninth author is incorrect. Rui Wu is not affiliated with #8 but with #7: Department of Infectious Diseases, Hangzhou Xixi Hospital, Hangzhou Sixth People's Hospital, Hangzhou Xixi Hospital Affiliated to Zhejiang Chinese Medical University, Hangzhou, Zhejiang, China.
