# Supplementary material for: Clinical efficacy of empiric antibiotic therapy in Chinese patients with cirrhosis and bacterial infections: A multicenter study
Source: PLoS One. 2026 Sep 15;21(9):e0357491. doi: 10.1371/journal.pone.0357491 (PMC13577579; doi:10.1371/journal.pone.0357491)
Supplement: Supplementary file 1 [file pone.0357491.s001.docx]

**Supplementary Appendix**

This appendix has been provided by the authors to give readers additional information about their work.

Supplement to: Clinical efficacy of empirical antibiotic therapy in Chinese patients with cirrhosis and bacterial infections: a multicenter study

**Supplementary Methods**

**Patients were excluded if any of the following applied:**

(1) presence of hepatocellular carcinoma; (2) extrahepatic malignant disease; (3) severe extrahepatic comorbidities, such as congestive heart failure (New York Heart Association class ≥ III), chronic obstructive pulmonary disease (Global Initiative for Chronic Obstructive Lung Disease stage ≥ III), or chronic kidney disease requiring renal replacement therapy; (4) prior solid organ transplantation; (5) documented human immunodeficiency virus infection; or (6) treatment with immunosuppressive agents (other than corticosteroids used for liver-related indications) within 1 month before hospital admission.

**Data collection**

We used a predefined standardized case report form to extract data from the electronic medical record systems of all participating centers. At the time of index infection, we collected demographic characteristics, microbiological findings, initial empiric antibiotic regimens, and subsequent antibiotic escalation. In addition, inflammatory markers, hemodynamic parameters and measures of liver disease severity were recorded. During follow-up, we documented the occurrence of new infections and organ failures (need for vasopressors, renal replacement therapy or mechanical ventilation), as well as transfer to the intensive care unit. In patients who developed a second infection during the same hospitalization, repeat microbiological cultures and antimicrobial susceptibility testing were performed.

Pre-admission information was also recorded and included: (1) antibiotic exposure within the preceding 3 months; (2) recent use (within 1 month) of medications such as rifaximin, β-blockers, proton pump inhibitors (PPIs) or quinolones for prophylaxis; (3) a history of invasive procedures during the previous 3 months (e.g. surgery, central venous catheter placement, indwelling urinary catheter or paracentesis); (4) admission to an intensive care unit within the week before the index hospitalization; and (5) any documented infection within the 3 months prior to admission.

**The diagnostic criteria for the various types of bacterial infection were defined as follows(1):**

(1) Pneumonia was diagnosed in the presence of a new pulmonary infiltrate on chest imaging together with at least one of the following: respiratory symptoms (e.g. cough, sputum production, dyspnea or pleuritic chest pain), abnormal chest auscultation (rales or crackles), fever with or without chills, or a white blood cell (WBC) count >10,000/mm³ or <4,000/mm³.

(2) Spontaneous bacterial peritonitis (SBP) was defined as an ascitic fluid polymorphonuclear (PMN) cell count ≥250 cells/mm³, irrespective of ascitic fluid culture results, in the absence of a surgically treatable intra-abdominal source of infection.

(3) Bacteremia was defined as at least one positive blood culture for a pathogenic organism, with or without an identifiable primary focus of infection.

(4) Urinary tract infection required pyuria (urine WBC >15 cells per high-power field) in combination with a positive urine culture and symptoms of urinary tract irritation (such as dysuria, frequency or urgency).

(5) Skin and soft-tissue infection was diagnosed in patients with clinical cellulitis accompanied by fever and leukocytosis.

(6) Other bacterial infections included microbiologically or clinically documented infections of the biliary tract, liver (e.g. liver abscess), central nervous system (e.g. purulent meningitis), testes, intra-abdominal cavity or other well-defined infectious foci.

**The criteria of the EASL-Chronic Liver Failure (CLIF) Consortium(2):**

- No ACLF:

Any of the following:

1. No organ failure, or
2. Single non-kidney organ failure (liver, coagulation, circulation or lungs) with serum creatinine < 1.5 mg/dL and no hepatic encephalopathy, or
3. Single cerebral failure with serum creatinine < 1.5 mg/dL.

- ACLF grade 1:

Any of the following:

1. Single kidney failure, or
2. Single non-kidney organ failure (liver, coagulation, circulation or lungs) with serum creatinine 1.5-1.9 mg/dL and/or grade I-II hepatic encephalopathy, or
3. Single cerebral failure with serum creatinine 1.5-1.9 mg/dL.

- ACLF grade 2: Two organ failures.
- ACLF grade 3: Three or more organ failures.

**Hepatic encephalopathy was graded according to the West-Haven criteria as follows(3):**

- Grade 0: No obvious mental status changes, normal consciousness and behavior, but minimal cognitive impairment may be detectable on specialized testing.
- Grade 1: Mild slowing of mental activity, slight lack of attention or concentration, subtle changes in behavior or personality, and/or mild disturbance of the sleep-wake cycle.
- Grade 2: Lethargy or drowsiness, more overt disorientation (especially to time), obvious personality or behavioral changes, inappropriate behavior, and asterixis.
- Grade 3: Marked somnolence but still arousable, confusion with gross disorientation, reduced response to verbal commands, possible agitation or combativeness.
- Grade 4: Coma, unresponsive to verbal or painful stimuli, often with decerebrate or decorticate posturing.

**The diagnosis criteria of scoring systems:**

- MELDs(4): 3.78 * ln [TB (mg/dL)] + 11.2 * ln (INR) + 9.57 * ln[serum creatinine (mg/dL)] + 6.434
- MELD-Nas(5): MELD + 1.59 (135–serum sodium)

**A positive quick Sequential Organ Failure Assessment (qSOFA) score(6)**:

Calculated based on three bedside variables-systolic blood pressure ≤100 mmHg, respiratory rate ≥22 breaths/min, and altered mentation (Glasgow Coma Scale <15)-with a score ≥2 indicating increased risk of poor outcome in patients with suspected infection.

**Systemic inflammatory response syndrome (SIRS)(7)**:

Diagnosed when at least two of the following criteria were met: (1) body temperature <36°C or >38°C; (2) heart rate >90 beats/min; (3) respiratory rate >20 breaths/min; (4) white blood cell count <4,000/mm³ or >12,000/mm³; or (5) proportion of immature neutrophils >10%.

**Septic shock(6):**

Diagnosed as sepsis with hypotension requiring vasopressors.

**The diagnosis MDR and XDR(6):**

Multidrug-resistant (MDR) bacteria were defined as isolates showing acquired resistance to at least one agent in three or more different antimicrobial classes, thereby substantially limiting first-line treatment options. Extensively drug-resistant (XDR) bacteria were defined as isolates that remain susceptible to no more than one or two antimicrobial classes, indicating resistance to almost all available antibiotic categories and leaving very few therapeutic alternatives.

**Supplementary Table 1. EASL empirical antibiotic treatment recommendations for community-acquired and nosocomial bacterial infections in cirrhosis(8)**

| Type of infection | Community-acquired infections | Nosocomial infections |
| --- | --- | --- |
| SBP, SBE, and spontaneous bacteremia | Cefotaxime or ceftriaxone  or amoxicillin-clavulanic acid | Piperacillin-tazobactam^†^  or meropenem^‡^ ± glycopeptide^§^ |
| UTI | | |
| Uncomplicated | Ciprofloxacin or cotrimoxazole | Nitrofurantoin  or fosfomycin |
| If sepsis | Cefotaxime  or ceftriaxone  or amoxicillin-clavulanic acid | Piperacillin-tazobactam^†^  or meropenem^‡^ ± glycopeptide^§^ |
| Pneumonia | Amoxicillin-clavulanic acid  or ceftriaxone + macrolide  or levofloxacin  or moxifloxacin | Piperacillin-tazobactam^†^  or meropenem-ceftazidime + ciprofloxacin ± glycopeptide^§^ should be added in patients with risk factors for MRSA^¶^ |
| Cellulitis | Amoxicillin-clavulanic acid  or ceftriaxone + oxacillin | Meropenem-ceftazidime^††^ + oxacillin  or glycopeptides^§^ |

Abbreviations: EASL, European Association for the Study of the Liver; SBP, spontaneous bacterial peritonitis; SBE, spontaneous bacterial empyema; UTI, urinary tract infection; MRSA, methicillin-resistant Staphylococcus aureus.

† In areas with a low prevalence of multidrug resistant bacteria.

‡ To cover extended-spectrum b-lactamase (ESBL)-producing Enterobacteriaceae.

§ Intravenous vancomycin or teicoplanin in areas with a high prevalence of MRSA and VSE. Glycopeptides must be replaced by linezolid in areas with a high prevalence of VRE.

¶ Ventilator-associated pneumonia, prior antibiotic use, and nasal MRSA carriage.

†† Antibiotics active against Pseudomonas aeruginosa.

**Supplementary Table 2. Comparison of patients’ characteristics according to the clinical response to empirical antibiotic treatment**

| Variable | Clinical response  (N = 913) | No clinical response  (N = 488) | *p* value |
| --- | --- | --- | --- |
| Age (y), mean (SD) | 62±13 | 61±14 | 0.119 |
| Sex (male), n (%) | 570(62.4%) | 324(66.4%) | 0.141 |
| Etiology of cirrhosis, n (%) | | | |
| Alcohol | 154(16.9%) | 96(19.7%) | 0.032 |
| HBV | 417(45.7%) | 211(43.2%) |  |
| PBC | 64(7.0%) | 25(5.1%) |  |
| AIH | 42(4.6%) | 30(6.1%) |  |
| Steatotic liver disease | 47(5.1%) | 30(6.1%) |  |
| Schistosome | 84(9.2%) | 26(5.3%) |  |
| Others | 105(11.5%) | 70(14.3%) |  |
| Comorbidities | | | |
| Hypertension | 248(27.2%) | 117(24.0%) | 0.195 |
| Diabetes | 224(24.5%) | 125(25.6%) | 0.597 |
| Past medical history | | | |
| TIPS | 33(3.6%) | 19(3.9%) | 0.792 |
| Infection in the past | 325(35.6%) | 191(39.1%) | 0.190 |
| Rifaximin use in the past month | 17(1.9%) | 7(1.4%) | 0.557 |
| β-blockers use in the past month | 42(4.6%) | 21(4.3%) | 0.798 |
| Proton pump inhibitors use in the past month | 172(18.8%) | 92(18.9%) | 0.995 |
| Quinolone antibiotics use in the past month | 49(5.4%) | 26(5.3%) | 0.975 |
| Antibiotic use in the past three months | 196(21.5%) | 124(25.4%) | 0.094 |
| Invasive procedures in the past month | 129(14.1%) | 87(17.8%) | 0.068 |
| Admitted to ICU in the past week | 9(1.0%) | 13(2.7%) | 0.016 |
| Mean arterial pressure (mm Hg), mean (SD) | 84±14 | 80±14 | <0.001 |
| Heart rate (beat/min), mean (SD) | 91±15 | 101±18 | <0.001 |
| Respiratory rate (breath/min), mean (SD) | 20±4 | 22±8 | <0.001 |
| WBC, *109/L, mean (SD) | 7.3±5.7 | 9.4±7.2 | <0.001 |
| NLR | 7.1±10.7 | 12.8±15.6 | <0.001 |
| CRP(mg/L), mean (SD) | 42.7±49.8 | 61.0±59.9 | <0.001 |
| Serum creatinine (umol/L), mean (SD) | 90.2±60.2 | 115.3±95.1 | <0.001 |
| Serum bilirubin (mg/dL), mean (SD) | 65.7±86.2 | 125.5±143.3 | <0.001 |
| INR, mean (SD) | 2.0±9.0 | 1.9±1.1 | 0.338 |
| Serum albumin (g/dL), mean (SD) | 29.7±6.6 | 27.5±5.9 | <0.001 |
| Serum sodium (mmol/L), mean (SD) | 136.5±5.0 | 135.1±6.2 | <0.001 |
| Ascites, n (%) | 395(45.4%) | 244(52.6%) | 0.016 |
| HE, n (%) | 134(14.7%) | 136(27.9%) | <0.001 |
| Severity scores | | | |
| MELD score, mean (SD) | 15.2±7.0 | 20.1±8.8 | <0.001 |
| MELD-Na score, mean (SD) | 17.2±9.1 | 23.7±11.6 | <0.001 |
| Acute on chronic liver failure grade, n (%) | | | |
| No ACLF | 799(87.5%) | 317(65.0%) | <0.001 |
| ACLF-1 | 64(7.0%) | 46(9.4%) |  |
| ACLF-2 | 41(4.5%) | 60(12.3%) |  |
| ACLF-3 | 9(1.0%) | 65(13.3%) |  |
| Organ failure | | | |
| Liver failure, n (%) | 62(6.8%) | 98(20.1%) | <0.001 |
| Coagulation failure, n (%) | 28(3.1%) | 59(21.1%) | <0.001 |
| Renal failure, n (%) | 59(6.5%) | 64(13.1%) | <0.001 |
| Brain failure, n (%) | 48(5.3%) | 79(16.2%) | <0.001 |
| Circulatory failure, n (%) | 51(5.6%) | 107(21.9%) | <0.001 |
| Respiratory failure, n (%) | 15(1.6%) | 67(13.7%) | <0.001 |
| Site of infection, n (%) |  |  |  |
| Urinary tract infection | 86(9.4%) | 40(8.2%) | 0.446 |
| Spontaneous bacterial peritonitis | 205(22.5%) | 112(23.0%) | 0.832 |
| Pneumonia | 267(29.2%) | 168(37.5%) | 0.046 |
| Spontaneous bacteremia | 119(13.0%) | 114(23.4%) | <0.001 |
| Skin and soft tissue infection | 61(6.7%) | 34(7.0%) | 0.839 |
| Intestinal infection | 30(3.3%) | 13(3.4%) | 0.520 |
| Cholecystitis/Chosalitis | 84(9.2%) | 33(8.5%) | 0.116 |
| Other abdominal infection | 42(4.6%) | 24(6.2%) | 0.795 |
| Other | 127(13.9%) | 71(14.5%) | 0.744 |
| Type of infection, n (%) |  |  |  |
| Community-acquired | 797(87.3%) | 414(84.8%) | 0.200 |
| Nosocomial | 116(12.7%) | 74(15.2%) |  |
| Severity of infection, n (%) |  |  |  |
| SIRS, n (%) | 429(47.0%) | 337(69.1%) | <0.001 |
| qSOFA, n (%) | 36(3.9%) | 66(13.5%) | <0.001 |
| Septic shock, n (%) | 30(3.3%) | 86(17.6%) | <0.001 |
| MDR, n (%) | 115(39.2%) | 118(49.6%) | <0.001 |
| XDR, n (%) | 4(1.1%) | 12(3.3%) | 0.041 |
| Isolates per patient, n (%) |  |  |  |
| 0 | 585(64.1%) | 214(43.9%) | <0.001 |
| 1 | 289(31.7%) | 202(41.4%) |  |
| >1 | 39(4.3%) | 72(14.8%) |  |
| Type of strains isolated, n (%) |  |  |  |
| Gram-negative | 226(61.4%) | 204(55.9%) | 0.152 |
| Gram-positive | 101(27.4%) | 104(28.5%) |  |
| Fungi | 41(11.1%) | 57(15.7%) |  |
| Most frequently isolated bacteria, n (%) | | | |
| *Escherichia coli* | 79(21.5%) | 59(16.2%) | 0.066 |
| *Klebsiella pneumoniae* | 67(18.2%) | 61(16.7%) | 0.594 |
| *Staphylococcus aureus* | 22(6.0%) | 33(9.0%) | 0.116 |
| *Acinetobacter baumannii* | 14(3.8%) | 16(4.4%) | 0.692 |
| *Enterococcus faecium* | 8(2.2%) | 20(5.5%) | 0.020 |
| *Enterococcus faecalis* | 6(1.6%) | 5(1.4%) | 0.772 |
| *Pseudomonas aeruginosa* | 13(3.5%) | 9(2.5%) | 0.397 |
| *Streptococcus* | 27(7.3%) | 16(4.4%) | 0.089 |
| *Candida* | 33(9.0%) | 35(9.6%) | 0.772 |
| *Aspergillus* | 6(1.6%) | 14(3.8%) | 0.067 |
| Types of empirical antibiotics | | | |
| Classic β-lactams plus β-lactamase inhibitors ^†^ | 56(6.2%) | 21(4.3%) | 0.152 |
| Specific β-lactamase inhibitors^‡^ | 376(41.4%) | 189(38.9%) | 0.372 |
| Third-generation cephalosporin antibiotics | 172(18.9%) | 108(22.2%) | 0.142 |
| Quinolone antibiotics | 97(10.7%) | 57(11.7%) | 0.547 |
| Carbapenem antibiotics | 155(17.1%) | 90(18.5%) | 0.491 |
| Glycopeptide antibiotics | 6(0.7%) | 3(0.6%) | 0.925 |
| Linezolid | 2(0.2%) | 0(0.0%) | 0.546 |
| Other | 45(5.0%) | 18(3.7%) | 0.286 |
| Antibiotic resistance |  |  |  |
| Resistance | 203(46.8%) | 182(59.3%) | 0.003 |
| Non-resistant | 82(18.9%) | 47(15.3%) |  |
| Not test | 149(34.3%) | 78(25.4%) |  |
| Type of empirical antibiotic strategies | | | |
| Classical | 305(33.4%) | 174(35.7%) | 0.406 |
| MDR coverage | 607(66.6%) | 314(64.3%) |  |
| Change of antibiotic treatment | | | |
| Escalation | 0(0.0%) | 426(87.3%) | <0.001 |
| De-escalation | 114(12.5%) | 3(0.6%) |  |
| No change | 799(87.5%) | 59(12.1%) |  |
| LT-free mortality | | | |
| 28-day | 28(3.3%) | 104(22.6%) | <0.001 |
| 90-day | 66(7.8%) | 151(33.3%) | <0.001 |

Note: Data presented as number (percentage) for categorical variables and mean±SD for continuous variables.

Abbreviations: HBV, hepatitis B virus; PBC, primary biliary cholangitis; AIH, autoimmune hepatitis; TIPS, transjugular intrahepatic portosystemic shunt; WBC, white blood cell; NLR, neutrophil-to-lymphocyte ratio; CRP, C-reactive protein; INR, international normalized ratio; HE, hepatic encephalopathy; MELD, model for end-stage liver disease; MELD-Na, MELD-sodium; ACLF, acute on chronic liver failure; SIRS, systemic inflammatory response syndrome; qSOFA, quick sequential organ failure assessment; MDR, multiple drug resistance; XDR, extensively drug-resistant; LT, liver transplant.

† Amoxicillin–clavulanic acid or ampicillin–sulbactam

‡ Piperacillin-tazobactam and cefoperazone-sulbactam

**Supplementary Table 3. Independent predictors of lack of clinical response to empirical antibiotic treatment**

| Variable | OR (95% CI) | *p* value |
| --- | --- | --- |
| Age (y) | 0.995(0.987-1.003) | 0.233 |
| Sex (male) | 0.841(0.668-1.059) | 0.142 |
| Etiology of cirrhosis |  |  |
| Alcohol | 1.207(0.910-1.601) | 0.192 |
| HBV | 0.906(0.726-1.131) | 0.382 |
| PBC | 0.716(0.445-1.153) | 0.169 |
| AIH | 1.358(0.839-2.200) | 0.213 |
| Steatotic liver disease | 1.207(0.753-1.935) | 0.435 |
| Schistosome | 0.855(0.553-1.175) | 0.061 |
| Infection in the past | 1.158(923-1.452) | 0.206 |
| Admitted to ICU in the past week | 2.749(1.167-6.478) | 0.021 |
| Mean arterial pressure (mm Hg) | 0.978(0.970-0.986) | <0.001 |
| Heart rate (beat/min) | 1.038(1.030-1.046) | <0.001 |
| Respiratory rate (breath/min) | 1.115(1.078-1.153) | <0.001 |
| WBC, *109/L | 1.054(1.035-1.073) | <0.001 |
| NLR | 1.038(1.027-1.049) | <0.001 |
| CRP(mg/L) | 1.006(1.004-1.008) | <0.001 |
| Serum creatinine (umol/L) | 1.005(1.003-1.006) | <0.001 |
| Serum bilirubin (mg/dL) | 1.005(1.004-1.006) | <0.001 |
| INR | 0.997(0.981-1.014) | 0.758 |
| Serum albumin (g/dL) | 0.943(0.925-0.961) | <0.001 |
| Serum sodium (mmol/L) | 0.954(0.935-0.974) | <0.001 |
| Ascites | 1.337(1.066-1.675) | 0.012 |
| HE | 2.246(1.715-2.942) | <0.001 |
| Severity scores | | |
| MELD score | 1.086(1.069-1.103) | <0.001 |
| MELD-Na score | 1.063(1.051-1.076) | <0.001 |
| ACLF grade | 2.198(1.902-2.541) | <0.001 |
| Organ failure | | |
| Liver failure | 3.449(2.455-4.845) | <0.001 |
| Coagulation failure | 4.313(2.711-6.861) | <0.001 |
| Renal failure | 2.182(1.504-3.167) | <0.001 |
| Brain failure | 3.481(2.386-5.077) | <0.001 |
| Circulatory failure | 4.747(3.330-6.767) | <0.001 |
| Respiratory failure | 9.527(5.379-16.876) | <0.001 |
| SIRS | 2.518(1.997-3.715) | <0.001 |
| qSOFA | 3.815(2.500-5.820) | <0.001 |
| Septic shock | 6.297(4.088-9.699) | <0.001 |
| Type of infection | | |
| Community-acquired | 0.814(0.594-1.116) | 0.201 |
| Nosocomial | 1.228(0.896-1.683) | 0.201 |
| MDR | 1.522(1.077-2.151) | 0.017 |
| XDR | 0.323(0.103-1.012) | 0.052 |

Note: Data presented as number (percentage) for categorical variables and mean±SD for continuous variables.

Abbreviations: HBV, hepatitis B virus; PBC, primary biliary cholangitis; AIH, autoimmune hepatitis; TIPS, transjugular intrahepatic portosystemic shunt; WBC, white blood cell; NLR, neutrophil-to-lymphocyte ratio; CRP, C-reactive protein; INR, international normalized ratio; HE, hepatic encephalopathy; MELD, model for end-stage liver disease; MELD-Na, MELD-sodium; ACLF, acute on chronic liver failure.

**Supplementary Table 4. Clinical response to empirical antibiotic therapy in culture-positive and culture-negative infections**

| Variable | Clinical response | | | No clinical response | | |
| --- | --- | --- | --- | --- | --- | --- |
|  | culture-positive  (n=328) | culture-negative  (n=397) | *p* value | culture-positive  (n=274) | culture-negative  (n=165) | *p* value |
| Site of infection, n (%) | | | | | | |
| Urinary tract infection | 81(25.0%) | 1(0.3%) | <0.001 | 1(0.4%) | 1(0.6%) | 0.716 |
| Spontaneous bacterial peritonitis | 46(14.0%) | 113(28.5%) | <0.001 | 113(41.2%) | 48(29.1%) | 0.268 |
| Pneumonia | 68(20.7%) | 127(32.0%) | <0.001 | 127(46.4%) | 55(33.3%) | 0.007 |
| Spontaneous bacteremia | 119(36.3%) | 0(0.0%) | <0.001 | 0(0.0%) | 1(0.6%) | 0.197 |
| Skin and soft tissue infection | 14(4.3%) | 31(7.8%) | 0.049 | 31(11.3%) | 15(9.1%) | 0.461 |
| Intestinal infection | 26(7.9%) | 4(1.0%) | <0.001 | 4(1.5%) | 0(0.0%) | 0.170 |
| Cholecystitis/Chosalitis | 16(4.9%) | 20(5.0%) | 0.921 | 20(7.3%) | 8(4.8%) | 0.309 |
| Other abdominal infection | 15(4.6%) | 21(6.8%) | 0.658 | 21(7.7%) | 7(4.2%) | 0.155 |
| Other | 18(5.5%) | 94(23.7%) | <0.001 | 94(34.3%) | 38(23.0%) | 0.013 |
| Type of infection, n (%) | | | | | | |
| Community-acquired | 272(82.9%) | 355(89.4%) | 0.011 | 230(83.9%) | 140(84.8%) | 0.800 |
| Nosocomial | 56(17.1%) | 42(10.6%) |  | 44(16.1%) | 25(15.2%) |  |
| Severity of infection, n (%) | | | | | | |
| SIRS, n (%) | 162(49.4%) | 199(50.1%) | 0.844 | 192(70.1%) | 112(67.9%) | 0.629 |
| qSOFA, n (%) | 14(4.3%) | 15(3.8%) | 0.738 | 38(13.9%) | 21(12.7%) | 0.734 |
| Septic shock, n (%) | 19(5.8%) | 7(1.8%) | 0.004 | 55(20.1%) | 22(13.3%) | 0.072 |
| Types of empirical antibiotics | | | | | | |
| Classic β-lactams plus β-lactamase inhibitors ^†^ | 14(4.3%) | 25(6.3%) | 0.228 | 11(4.0%) | 7(4.2%) | 0.907 |
| Specific β-lactamase inhibitors^‡^ | 128(39.3%) | 181(45.7%) | 0.075 | 108(39.7%) | 68(41.2%) | 0.710 |
| Third-generation cephalosporin antibiotics | 50(15.3%) | 73(18.4%) | 0.262 | 55(20.2%) | 34(20.6%) | 0.893 |
| Quinolone antibiotics | 33(10.1%) | 37(9.3%) | 0.737 | 30(11.0%) | 22(13.3%) | 0.454 |
| Carbapenem antibiotics | 80(24.5%) | 56(14.1%) | <0.001 | 56(20.6%) | 28(17.0%) | 0.371 |
| Glycopeptide antibiotics | 6(1.8%) | 0(0.0%) | 0.008 | 2(0.7%) | 0(0.0%) | 0.530 |
| Linezolid | 2(0.6%) | 0(0.0%) | 0.204 | 0(0.0%) | 0(0.0%) | - |
| Other | 13(4.0% ) | 24(6.1%) | 0.237 | 10(3.7%) | 6(3.6%) | 0.994 |
| Type of empirical antibiotic strategies | | | | | | |
| Classical | 95(29.0%) | 123(31.0%) | 0.555 | 91(33.2%) | 57(34.5%) | 0.775 |
| MDR coverage | 233(71.0%) | 274(69.0%) |  | 183(66.8%) | 108(65.5%) |  |
| Change of antibiotic treatment | | | | | | |
| Escalation | 0(0.0%) | 0(0.0%) | 0.085 | 238(86.9%) | 146(88.5%) | 0.536 |
| De-escalation | 54(16.5%) | 47(11.8%) |  | 1(0.4%) | 2(1.2%) |  |
| No change | 274(83.5%) | 350(88.2%) |  | 35(12.8%) | 17(10.3%) |  |

Note: Data presented as number (percentage) for categorical variables.

Abbreviations: SIRS, systemic inflammatory response syndrome; qSOFA, quick sequential organ failure assessment; MDR, multiple drug resistance;

† Amoxicillin–clavulanic acid or ampicillin–sulbactam

‡ Piperacillin-tazobactam and cefoperazone-sulbactam

**Supplementary Table 5. Clinical and microbiological characteristics of secondary infections stratified by clinical response to initial empiric antibiotics and to antibiotics for secondary infection**

| Variable | Second Response (N = 98) | | | | | |
| --- | --- | --- | --- | --- | --- | --- |
|  | Clinical response to empirical antibiotics  (N = 30) | No clinical response to empirical antibiotics  (N = 68) | *p* value | Clinical response to antibiotics after second infection  (N= 70 ) | No clinical response to antibiotics after second infection  (N = 28) | *p* value |
| Site of secondary infection, n (%) | | | | | | |
| Urinary tract infection | 3(10.0%) | 18(26.5%) | 0.067 | 13(18.6%) | 8(28.6%) | 0.276 |
| Spontaneous bacterial peritonitis | 0(0.0%) | 8(11.8%) | 0.102 | 6(8.6%) | 2(7.1%) | 0.816 |
| Pneumonia | 8(26.7%) | 17(25.0%) | 0.862 | 15(21.4%) | 10(35.7%) | 0.143 |
| Spontaneous bacteremia | 8(26.7%) | 13(19.1%) | 0.401 | 16(22.9%) | 5(17.9%) | 0.586 |
| Skin and soft tissue infection | 4(13.3%) | 3(4.4%) | 0.214 | 6(8.6%) | 1(3.6%) | 0.385 |
| Intestinal infection | 2(6.7%) | 8(11.8%) | 0.442 | 8(11.4%) | 2(7.1%) | 0.527 |
| Cholecystitis/Chosalitis | 3(10.0%) | 0(0.0%) | 0.027 | 2(2.9%) | 1(3.6%) | 0.853 |
| Other abdominal infection | 1(3.3%) | 3(4.4%) | 0.804 | 4(5.7%) | 0(0.0%) | 0.323 |
| Other | 4(13.3%) | 10(14.7%) | 0.858 | 10(14.3%) | 4(14.3%) | 1.000 |
| Patients with positive cultures of secondary infection, n (%) | 20(83.3%) | 63(88.3%) | 0.866 | 58(84.1%) | 25(78.1%) | 0.469 |
| Antibiotic resistance of secondary infection, n (%) | 12(36.4%) | 49(87.5%) | 0.003 | 41(87.2%) | 20(87.0%) | 0.974 |
| MDR of secondary infection, n (%) | 11(33.3%) | 34(42.5%) | 0.365 | 31(38.8%) | 14(42.4%) | 0.717 |

Note: Data presented as number (percentage) for categorical variables.

Abbreviations: MDR, multiple drug resistance;

**Supplementary Table 6 Demographic, clinical and laboratory characteristics of 28-day survivors vs non-survivors**

| Variable | Survivors  (n=1158) | Nonsurvivors  (n=132) | *p* value |
| --- | --- | --- | --- |
| Age (y), mean±SD | 61.8±13.1 | 60.9±14.6 | 0.420 |
| Sex (male), n (%) | 751(63.4%) | 85(64.4%) | 0.917 |
| Etiology of cirrhosis, n (%) | | | |
| Alcohol | 219(18.5%) | 18(13.6%) | 0.516 |
| HBV | 524(44.2%) | 61(46.2%) |  |
| PBC | 80(6.8%) | 6(4.5%) |  |
| AIH | 61(5.1%) | 9(6.8%) |  |
| Steatotic liver disease | 59(5.0%) | 10(7.6%) |  |
| Schistosome | 94(7.9%) | 9(6.8%) |  |
| Others | 148(12.5%) | 19(14.4%) |  |
| Comorbidities | | | |
| Hypertension | 312(26.3%) | 33(25.0%) | 0.633 |
| Diabetes | 293(24.7%) | 35(26.5%) | 0.762 |
| Past medical history | | | |
| TIPS | 40(3.4%) | 7(5.3%) | 0.283 |
| Infection in the past | 431(36.5%) | 54(40.9%) | 0.407 |
| Rifaximin use in the past month | 20(1.7%) | 1(0.8%) | 0.404 |
| β-blockers use in the past month | 54(4.6%) | 7(5.3%) | 0.743 |
| Proton pump inhibitors use in the past month | 228(19.2%) | 20(15.2%) | 0.210 |
| Quinolone antibiotics use in the past month | 66(5.6%) | 8(6.1%) | 0.866 |
| Antibiotic use in the past three months | 269(22.7%) | 36(27.3%) | 0.300 |
| Invasive procedures in the past month | 183(15.4%) | 26(19.7%) | 0.250 |
| Admitted to ICU in the past week | 17(1.4%) | 5(3.8%) | 0.051 |
| Mean arterial pressure (mm Hg), mean±SD | 83±14 | 78±17 | <0.001 |
| Heart rate (beat/min), mean±SD | 93±16 | 106±22 | <0.001 |
| Respiratory rate (breath/min), mean (SD) | 21±5 | 23±6 | <0.001 |
| WBC, *109/L, mean±SD | 7.6±5.7 | 12.2±8.8 | <0.001 |
| NLR | 8.1±11.6 | 17.7±18.1 | <0.001 |
| CRP(mg/L), mean±SD | 47.4±52.8 | 66.4±66.8 | <0.001 |
| Serum creatinine (umol/L), mean±SD | 93.5±66.0 | 147.9±110.9 | <0.001 |
| Serum bilirubin (umol/L), mean±SD | 73.2±96.1 | 187.6±165.5 | <0.001 |
| INR, mean±SD | 1.9±7.9 | 2.4±1.5 | 0.526 |
| Serum albumin (g/dL), mean±SD | 29.3±6.1 | 26.7±8.4 | <0.001 |
| Serum sodium (mmol/L), mean±SD | 136.3±5.0 | 134.1±7.8 | <0.001 |
| Ascites, n (%) 1131 125 | 531(46.9%) | 71(56.8%) | 0.083 |
| HE, n (%) | 200(16.9%) | 61(46.2%) | <0.001 |
| Severity scores | | | |
| MELD score, mean±SD | 15.8±7.2 | 25.9±9.4 | <0.001 |
| MELD-Na score, mean±SD | 18.1±9.4 | 31.1±12.8 | <0.001 |
| Acute on chronic liver failure grade, n (%) | | | |
| No ACLF | 994(83.9%) | 51(38.6%) | <0.001 |
| ACLF-1 | 84(7.1%) | 15(11.4%) |  |
| ACLF-2 | 79(6.7%) | 20(15.2%) |  |
| ACLF-3 | 28(2.4%) | 46(34.8%) |  |
| Organ failure | | | |
| Liver failure, n (%) | 100(8.4%) | 46(34.8%) | <0.001 |
| Coagulation failure, n (%) | 51(4.3%) | 31(23.5%) | <0.001 |
| Renal failure, n (%) | 85(7.2%) | 31(23.5%) | <0.001 |
| Brain failure, n (%) | 80(6.8%) | 45(34.1%) | <0.001 |
| Circulatory failure, n (%) | 105(8.9%) | 51(38.6%) | <0.001 |
| Respiratory failure, n (%) | 43(3.6%) | 39(29.5%) | <0.001 |
| Urinary tract infection | | | |
| Spontaneous bacterial peritonitis | 272(23.5%) | 30(22.7%) | 0.845 |
| Pneumonia | 351(30.3%) | 54(40.9%) | 0.013 |
| Spontaneous bacteremia | 193(16.7%) | 26(19.7%) | 0.380 |
| Skin and soft tissue infection | 72(6.2%) | 15(11.4%) | 0.026 |
| Intestinal infection | 38(3.3%) | 1(0.8%) | 0.109 |
| Cholecystitis/Chosalitis | 103(8.9%) | 3(2.3%) | 0.009 |
| Other abdominal infection | 57(4.9%) | 7(5.3%) | 0.849 |
| Other | 181(15.6%) | 5(1.8%) | <0.001 |
| Type of infection, n (%) | | | |
| Community-acquired | 1027(86.7%) | 112(84.8%) | 0.562 |
| Nosocomial | 158(13.3%) | 20(15.2%) |  |
| Severity of infection, n (%) | | | |
| SIRS, n (%) | 631(53.2%) | 88(66.7%) | 0.008 |
| qSOFA, n (%) | 61(5.1%) | 39(29.5%) | <0.001 |
| Septic shock, n (%) | 70(5.9%) | 45(34.1%) | <0.001 |
| MDR, n (%) | 211(18.2%) | 29(22.0%) | 0.294 |
| XDR, n (%) | 10(0.9%) | 4(3.0%) | 0.023 |
| Clinical response to empirical antibiotic treatment, n (%) | 828(69.9%) | 28(21.2%) | 0.246 |
| Isolates per patient, n (%) | | | |
| 0 | 697(58.8%) | 58(43.9%) | <0.001 |
| 1 | 399(33.7%) | 54(40.9%) |  |
| >1 | 89(7.5%) | 20(15.2%) |  |
| Change of antibiotic treatment | | | |
| Escalation | 336(28.4%) | 63(47.7%) | <0.001 |
| De-escalation | 105(8.9%) | 5(3.8%) |  |
| No change | 744(62.8%) | 64(48.5%) |  |
| Second infections, n (%) | 79(6.7%) | 13(9.8%) | 0.202 |
| Resolution of infection, n (%) | 828(69.9%) | 28(21.2%) | 0.201 |

Note: Data presented as number (percentage) for categorical variables and mean±SD for continuous variables.

Abbreviations: HBV, hepatitis B virus; PBC, primary biliary cholangitis; AIH, autoimmune hepatitis; TIPS, transjugular intrahepatic portosystemic shunt; WBC, white blood cell; NLR, neutrophil-to-lymphocyte ratio; CRP, C-reactive protein; INR, international normalized ratio; HE, hepatic encephalopathy; MELD, model for end-stage liver disease; MELD-Na, MELD-sodium; ACLF, acute on chronic liver failure; SIRS, systemic inflammatory response syndrome; qSOFA, quick sequential organ failure assessment; MDR, multiple drug resistance; XDR, extensively drug-resistant;

**Supplementary Table 7 Demographic, clinical and laboratory characteristics of 90-day survivors vs non-survivors**

| Variable | Survivors  (n=1079) | Nonsurvivors  (n=217) | *p* value |
| --- | --- | --- | --- |
| Age (y), mean±SD | 61.6±13.0 | 62.3±14.3 | 0.500 |
| Sex (male), n (%) | 695(64.4%) | 130(59.9%) | 0.208 |
| Etiology of cirrhosis, n (%) | | | |
| Alcohol | 199(18.4%) | 35(16.1%) | 0.096 |
| HBV | 485(44.9%) | 94(43.3%) |  |
| PBC | 68(6.3%) | 15(6.9%) |  |
| AIH | 48(4.4%) | 21(9.7%) |  |
| Steatotic liver disease | 55(5.1%) | 12(5.5%) |  |
| Schistosome | 87(8.1%) | 14(6.5%) |  |
| Others | 137(12.7%) | 26(12.0%) |  |
| Comorbidities | | | |
| Hypertension | 276(25.6%) | 64(29.5%) | 0.232 |
| Diabetes | 265(24.6%) | 58(26.7%) | 0.500 |
| Past medical history | | | |
| TIPS | 36(3.3%) | 11(5.1%) | 0.213 |
| Infection in the past | 382(35.5%) | 96(44.4%) | 0.014 |
| Rifaximin use in the past month | 16(1.5%) | 6(2.8%) | 0.182 |
| β-blockers use in the past month | 49(4.5%) | 12(5.5%) | 0.530 |
| Proton pump inhibitors use in the past month | 198(18.4%) | 47(21.7%) | 0.256 |
| Quinolone antibiotics use in the past month | 55(5.1%) | 17(7.8%) | 0.108 |
| Antibiotic use in the past three months | 236(21.9%) | 65(30.0%) | 0.010 |
| Invasive procedures in the past month | 161(14.9%) | 47(21.7%) | 0.014 |
| Admitted to ICU in the past week | 15(1.4%) | 7(3.2%) | 0.056 |
| Mean arterial pressure (mm Hg), mean±SD | 83±14 | 79±17 | <0.001 |
| Heart rate (beat/min), mean±SD | 93±15 | 102±21 | <0.001 |
| Respiratory rate (breath/min), mean (SD) | 21±5 | 22±6 | <0.001 |
| WBC, *109/L, mean±SD | 7.5±5.7 | 10.7±7.9 | <0.001 |
| NLR | 8.1±11.7 | 14.0±16.5 | <0.001 |
| CRP(mg/L), mean±SD | 47.2±52.6 | 60.9±63.8 | <0.001 |
| Serum creatinine (umol/L), mean±SD | 92.9±65.8 | 130.1±100.3 | <0.001 |
| Serum bilirubin (umol/L), mean±SD | 67.5±89.1 | 170.9±159.6 | <0.001 |
| INR, mean±SD | 1.9±8.3 | 2.1±1.3 | 0.810 |
| Serum albumin (g/dL), mean±SD | 29.4±6.2 | 27.1±7.2 | <0.001 |
| Serum sodium (mmol/L), mean±SD | 136.4±5.0 | 134.3±7.1 | <0.001 |
| Ascites, n (%) | 477(46.1%) | 119(58.6%) | 0.001 |
| HE, n (%) | 164(15.2%) | 95(43.8%) | <0.001 |
| Severity scores | | | |
| MELD score, mean±SD | 15.5±7.2 | 23.5±9.1 | <0.001 |
| MELD-Na score, mean±SD | 17.6±9.3 | 28.1±12.1 | <0.001 |
| Acute on chronic liver failure grade, n (%) | | | |
| No ACLF | 926(85.8%) | 102(47.0%) | <0.001 |
| ACLF-1 | 71(6.6%) | 26(12.0%) |  |
| ACLF-2 | 57(5.3%) | 40(18.4%) |  |
| ACLF-3 | 25(2.3%) | 49(22.6%) |  |
| Organ failure | | | |
| Liver failure, n (%) | 74(6.9%) | 70(32.3%) | <0.001 |
| Coagulation failure, n (%) | 43(4.0%) | 38(17.5%) | <0.001 |
| Renal failure, n (%) | 74(6.9%) | 41(18.9%) | <0.001 |
| Brain failure, n (%) | 63(5.8%) | 61(28.1%) | <0.001 |
| Circulatory failure, n (%) | 87(8.1%) | 68(31.3%) | <0.001 |
| Respiratory failure, n (%) | 40(3.7%) | 42(19.4%) | <0.001 |
| Urinary tract infection | | | |
| Spontaneous bacterial peritonitis | 229(21.2%) | 56(25.8%) | 0.137 |
| Pneumonia | 315(29.2%) | 72(33.2%) | 0.242 |
| Spontaneous bacteremia | 171(15.8%) | 41(18.9%) | 0.268 |
| Skin and soft tissue infection | 65(6.0%) | 20(9.2%) | 0.083 |
| Intestinal infection | 34(3.2%) | 4(1.8%) | 0.297 |
| Cholecystitis/Chosalitis | 92(8.5%) | 12(5.5%) | 0.138 |
| Other abdominal infection | 55(5.1%) | 6(2.8%) | 0.139 |
| Other | 162(15.0%) | 12(5.5%) | <0.001 |
| Type of infection, n (%) | | | |
| Community-acquired | 945(87.6%) | 176(81.1%) | 0.011 |
| Nosocomial | 134(12.4%) | 41(18.9%) |  |
| Severity of infection, n (%) | | | |
| SIRS, n (%) | 571(52.95) | 137(63.1%) | 0.006 |
| qSOFA, n (%) | 54(5.0%) | 45(24.9%) | <0.001 |
| Septic shock, n (%) | 61(5.7%) | 53(24.4%) | <0.001 |
| MDR, n (%) | 182(16.9%) | 45(22.1%) | 0.171 |
| XDR, n (%) | 10(0.9%) | 4(1.8%) | 0.233 |
| Clinical response to empirical antibiotic treatment, n (%) | 777(72.0%) | 66(30.4%) | <0.001 |
| Isolates per patient, n (%) | | | |
| 0 | 643(59.6%) | 98(45.2%) | <0.001 |
| 1 | 359(33.3%) | 88(40.6%) |  |
| >1 | 77(7.1%) | 31(14.3%) |  |
| Change of antibiotic treatment | | | |
| Escalation | 288(26.7%) | 103(47.5%) | <0.001 |
| De-escalation | 96(8.9%) | 13(6.0%) |  |
| No change | 695(64.4%) | 101(46.5%) |  |
| Second infections, n (%) | 61(5.7%) | 29(13.4%) | <0.001 |
| Resolution of infection, n (%) | 1026(95.1%) | 71(32.7%) | <0.001 |

Note: Data presented as number (percentage) for categorical variables and mean±SD for continuous variables.

Abbreviations: HBV, hepatitis B virus; PBC, primary biliary cholangitis; AIH, autoimmune hepatitis; TIPS, transjugular intrahepatic portosystemic shunt; WBC, white blood cell; NLR, neutrophil-to-lymphocyte ratio; CRP, C-reactive protein; INR, international normalized ratio; HE, hepatic encephalopathy; MELD, model for end-stage liver disease; MELD-Na, MELD-sodium; ACLF, acute on chronic liver failure; SIRS, systemic inflammatory response syndrome; qSOFA, quick sequential organ failure assessment; MDR, multiple drug resistance; XDR, extensively drug-resistant;

**Supplementary Table 8. Multivariable Cox regression analysis of independent predictors of 28-day and 90-day mortality**

| Variable | sHR | 95%CI | *P* Value |
| --- | --- | --- | --- |
| 28-day mortality | | | |
| Age | 1.020 | 1.006-1.034 | 0.005 |
| NLR | 1.012 | 1.003-1.022 | 0.008 |
| Serum albumin | 0.957 | 0.924-0.991 | 0.013 |
| Hepatic encephalopathy | 1.712 | 1.148-2.553 | 0.008 |
| MELDNa | 1.053 | 1.037-1.068 | <0.001 |
| SIRS | 0.676 | 0.434-1.052 | 0.083 |
| Septic shock | 2.674 | 1.668-4.287 | <0.001 |
| qSOFA | 1.853 | 1.150-2.985 | 0.011 |
| Clinical response | 4.200 | 2.673-6.601 | <0.001 |
| Pneumonia | 1.453 | 0.996-2.118 | 0.052 |
| 90-day mortality | | | |
| Age | 1.020 | 1.009-1.032 | <0.001 |
| Sex | 1.538 | 1.146-2.064 | 0.004 |
| Serum albumin | 0.963 | 0.938-0.990 | 0.007 |
| Hepatic encephalopathy | 1.759 | 1.262-2.451 | <0.001 |
| MELDNa | 1.048 | 1.034-1.062 | <0.001 |
| Septic shock | 1.431 | 0.937-2.186 | 0.097 |
| qSOFA | 1.432 | 0.943-2.174 | 0.092 |
| ACLF grade | | | |
| ACLF-1 | 1.603 | 0.990-2.595 | 0.055 |
| ACLF-2 | 1.868 | 1.170-2.983 | 0.009 |
| ACLF-3 | 1.982 | 1.096-3.583 | 0.024 |
| Clinical response | 3.160 | 2.308-4.326 | <0.001 |

Notes: Variables included in multivariate analysis: age, sex, Mean arterial pressure, C-reactive protein, leukocytes, total bilirubin, NLR, serum albumin, serum creatinine, serum sodium, ascites, hepatic encephalopathy, MELD, MELD-Na, SIRS, Septic shock, quick SOFA, MDR bacterial infections, ACLF grade, Clinical response to empirical treatment, Patients with positive cultures, nosocomial infections, pneumonia, spontaneous bacterial peritonitis, spontaneous bacteremia

Abbreviations: NLR, neutrophil-to-lymphocyte ratio; MELD-Na, MELD-sodium; SIRS, systemic inflammatory response syndrome; qSOFA, quick sequential organ failure assessment; ACLF, acute on chronic liver failure;

**Supplementary Table 9. Comparison of patient characteristics according to clinical response to empirical antibiotic therapy: present study versus global cohort**

| Variable | Clinical response | | No clinical response | |
| --- | --- | --- | --- | --- |
|  | China  (n=913) | Global  (n=788) | China  (n=488) | Global  (n=514) |
| Age (y), mean±SD | 62±13 | 57±13 | 61±14 | 55±13 |
| Sex (male), n (%) | 570(62%) | 516(66%) | 324(66%) | 382(74%) |
| Etiology of cirrhosis, n (%) | | | | |
| Alcohol | 154(17%) | 409(52%) | 96(20%) | 288(56%) |
| HCV | 0(0%) | 167(21%) | 0(0%) | 69(18%) |
| HBV | 417(46%) | 67(9%) | 211(43%) | 33(6%) |
| NASH | 47(5%) | 89(11%) | 30(6%) | 57(11%) |
| Others | 295(32%) | 149(19%) | 151(31%) | 87(17%) |
| Mean arterial pressure (mm Hg), mean±SD | 84±14 | 82±13 | 80±14 | 81±15 |
| Heart rate (beat/min), mean±SD | 91±15 | 87±17 | 101±18 | 90±17 |
| Respiratory rate (breath/min), mean±SD | 20±4 | 19±4 | 22±8 | 20±6 |
| Leukocytes (WBC; × 109/L), median (p25-p75) | 5.8(3.4-9.5) | 7.2(4.6-11.8) | 8.1(4.1-12.2) | 9.9(6.5-14.2) |
| C-reactive protein (mg/L), median (p25-p75) | 23(8-60) | 30(12-64) | 42(17-85) | 46(20-89) |
| Serum creatinine (mg/dL), median (p25-p75) | 0.8(0.7-1.1) | 1.0(0.8-1.7) | 1.0(0.8-1.5) | 1.3(0.9-2.1) |
| Serum bilirubin (mg/dL), median (p25-p75) | 2.0(1.0-4.6) | 3.3(1.6-6.9) | 3.7(1.7-9.4) | 4.4(2.0-10.0) |
| INR, median (p25-p75) | 1.3(1.2-1.6) | 1.6(1.3-2.0) | 1.6(1.3-2.0) | 1.7(1.4-2.2) |
| Serum albumin (g/dL), median (p25-p75) | 2.9(2.6-3.3) | 2.7(2.3-3.1) | 2.7(2.3-3.1) | 2.5(2.1-2.9) |
| Serum sodium (mmol/L), mean±SD | 136±5 | 133±6 | 135±6 | 132±8 |
| Ascites, n (%) | 395(45%) | 604(77%) | 244(53%) | 398(77%) |
| HE, n (%) | 134(15%) | 258(33%) | 136(28%) | 238(46%) |
| MELD score, mean±SD | 15±7 | 20±7 | 20±9 | 23±8 |
| MELD-Na score, mean±SD | 17±9 | 23±7 | 24±12 | 26±8 |
| Acute on chronic liver failure grade, n (%) | | | | |
| No ACLF | 799(88%) | 564(72%) | 317(65%) | 278(54%) |
| ACLF-1 | 64(7%) | 113(14%) | 46(9%) | 77(15%) |
| ACLF-2 | 41(5%) | 78(10%) | 60(12%) | 86(17%) |
| ACLF-3 | 9(1%) | 33(4%) | 65(13%) | 73(14%) |
| Liver failure, n (%) | 62(7%) | 102(13%) | 98(20%) | 109(21%) |
| Coagulation failure, n (%) | 28(3%) | 80(10%) | 59(21%) | 89(17%) |
| Renal failure, n (%) | 59(7%) | 137(17%) | 64(13%) | 145(28%) |
| Brain failure, n (%) | 48(5%) | 50(6%) | 79(16%) | 90(18%) |
| Circulatory failure, n (%) | 51(6%) | 77(10%) | 107(22%) | 97(19%) |
| Respiratory failure, n (%) | 15(2%) | 30(4%) | 67(14%) | 61(12%) |
| SIRS, n (%) | 429(47%) | 208(32%) | 337(69%) | 197(43%) |
| qSOFA, n (%) | 36(4%) | 121(18%) | 66(14%) | 134(29%) |
| Septic shock, n (%) | 30(3%) | 77(10%) | 86(18%) | 97(19%) |
| Site of infection, n (%) | | | | |
| Urinary tract infection | 86(9%) | 193(25%) | 40(8%) | 96(19%) |
| Spontaneous bacterial peritonitis | 205(23%) | 226(29%) | 112(23%) | 128(25%) |
| Pneumonia | 267(29%) | 108(14%) | 168(38%) | 134(26%) |
| Spontaneous bacteremia | 119(13%) | 64(8%) | 114(23%) | 36(7%) |
| Skin and soft tissue infection | 61(7%) | 60(8%) | 34(7%) | 41(8%) |
| Other | 283(31%) | 137(17%) | 141(29%) | 79(15%) |
| Type of infection, n (%) | | | | |
| Community-acquired | 797(87%) | 391(50%) | 414(85%) | 237(46%) |
| Nosocomial | 116(13%) | 179(23%) | 74(15%) | 157(31%) |
| Culture-positive infections, n (%) | 328(36%) | 431(55%) | 165(34%) | 309(60%) |
| MDR bacterial infections, n (%) | 115(39%) | 100(13%) | 118(50%) | 153(30%) |
| XDR bacterial infections, n (%) | 4(1%) | 19(2%) | 12(3%) | 43(8%) |
| Fungi, n (%) | 41(11%) | 9(1%) | 57(16%) | 15(3%) |
| Treatment with 2 or more antibiotics, n (%) | 39(4%) | 264(34%) | 57(16%) | 185(36%) |

Note: Data presented as number (percentage) for categorical variables and mean (SD) for parametric or median (interquartile range) for non-parametric continuous variables.

Abbreviation: HCV, hepatitis C virus; HBV, hepatitis B virus; NASH, nonalcoholic steatohepatitis; HE, hepatic encephalopathy; MELD, model for end-stage liver disease; MELD-Na, MELD-sodium; ACLF, acute on chronic liver failure; SIRS, systemic inflammatory response syndrome; qSOFA, quick sequential organ failure assessment; MDR, multiple drug resistance; XDR, extensively drug-resistant;

**Supplementary References**

1.	Yu X, Liu X, Tan W, Wang X, Zheng X, Huang Y, Chen J, et al. The clinical courses of HBV-related acute-on-chronic liver failure and a multi-state model to predict disease evolution. Hepatol Commun 2024;8.

2.	Moreau R, Jalan R, Gines P, Pavesi M, Angeli P, Cordoba J, Durand F, et al. Acute-on-chronic liver failure is a distinct syndrome that develops in patients with acute decompensation of cirrhosis. Gastroenterology 2013;144:1426-1437, 1437.e1421-1429.

3.	Riordan SM, Williams R. Treatment of hepatic encephalopathy. N Engl J Med 1997;337:473-479.

4.	Choudhury A, Jindal A, Maiwall R, Sharma MK, Sharma BC, Pamecha V, Mahtab M, et al. Liver failure determines the outcome in patients of acute-on-chronic liver failure (ACLF): comparison of APASL ACLF research consortium (AARC) and CLIF-SOFA models. Hepatol Int 2017;11:461-471.

5.	Devarbhavi H, Choudhury AK, Sharma MK, Maiwall R, Al Mahtab M, Rahman S, Chawla YK, et al. Drug-Induced Acute-on-Chronic Liver Failure in Asian Patients. Am J Gastroenterol 2019;114:929-937.

6.	Singer M, Deutschman CS, Seymour CW, Shankar-Hari M, Annane D, Bauer M, Bellomo R, et al. The Third International Consensus Definitions for Sepsis and Septic Shock (Sepsis-3). Jama 2016;315:801-810.

7.	Bone RC, Balk RA, Cerra FB, Dellinger RP, Fein AM, Knaus WA, Schein RM, et al. Definitions for sepsis and organ failure and guidelines for the use of innovative therapies in sepsis. The ACCP/SCCM Consensus Conference Committee. American College of Chest Physicians/Society of Critical Care Medicine. Chest 1992;101:1644-1655.

8.	Jalan R, Fernandez J, Wiest R, Schnabl B, Moreau R, Angeli P, Stadlbauer V, et al. Bacterial infections in cirrhosis: a position statement based on the EASL Special Conference 2013. J Hepatol 2014;60:1310-1324.
