# Supplementary material for: Clinical efficacy of empiric antibiotic therapy in Chinese patients with cirrhosis and bacterial infections: A multicenter study
Source: PLoS One. 2026 Sep 15;21(9):e0357491. doi: 10.1371/journal.pone.0357491 (PMC13577579; doi:10.1371/journal.pone.0357491)
Supplement: Supplementary file 2 [file pone.0357491.s003.docx]

Revision for “Clinical efficacy of empiric antibiotic therapy in Chinese patients with cirrhosis and bacterial infections: a multicenter study” (Manuscript ID: PONE-D-26-28291)

Dear Editor,

We appreciate the opportunity to respond to the reviewers’ comments and to improve our manuscript entitled, “Clinical efficacy of empiric antibiotic therapy in Chinese patients with cirrhosis and bacterial infections: a multicenter study.” We have addressed each comment by all reviewers as below, with changes underlined and highlighted by yellow color. We hope that the resubmitted manuscript is acceptable for publication.

Funding Statement：

This work was supported by the National Major Science and Technology Projects of China (2025ZD01906200), and the National Natural Science Foundation of China (82570717).

Role of Funder statement：

The funders had no role in study design, data collection and analysis, decision to publish, or preparation of the manuscript.

Sincerely,

Prof. Xia Yu, MD

Email: zjuyuxia@zju.edu.cn

State Key Laboratory for Diagnosis and Treatment of Infectious Diseases,

National Clinical Research Center for Infectious Diseases,

National Medical Center for Infectious Diseases,

Collaborative Innovation Center for Diagnosis and Treatment of Infectious Diseases, The First Affiliated Hospital, College of Medicine, Zhejiang University

**RESPONSES OF THE AUTHORS TO REVIEWER’S COMMENTS:**

**REVIEWER'S COMMENTS TO AUTHOR:**

Reviewer #1:

This multicenter retrospective study evaluates the clinical efficacy of empiric antibiotic therapy in Chinese patients with cirrhosis and bacterial or fungal infections. Based on a large cohort from 24 tertiary centers, the authors describe the rate of early clinical response, identify clinical and inflammatory predictors of non-response, and further examine the associations of treatment response and secondary infection with short-term mortality. Overall, the study addresses a clinically relevant question and provides useful real-world data for risk-adapted antibiotic management in cirrhosis. However, several issues might be further resolved.

1. Clinical response is a central exposure in this study and is strongly associated with 28-day and 90-day mortality. Since the median time to clinical response was 5 days, it would be helpful to clarify the exact time window used to define response and how early deaths before response assessment were handled. Please consider performing a sensitivity analysis treating clinical response as a time-dependent variable in the Cox model.

Response: Thank you for this important comment. Clinical response was not assessed at a single prespecified time point. Instead, it was evaluated longitudinally from the initiation of first-line empiric antibiotic therapy, which was defined as time zero, until hospital discharge or in-hospital death. During hospitalization, patients underwent repeated clinical assessment and laboratory testing according to routine clinical practice, generally at intervals of approximately 1-2 days. The date of clinical response was defined as the first date on which the patient met the prespecified clinical and laboratory response criteria.

In the primary analysis, patients were classified as responders if they demonstrated documented improvement in infection- or inflammation-related manifestations, inflammatory markers, and infection-related organ dysfunction during the index hospitalization. Patients who did not achieve a documented response before hospital discharge, or who experienced persistent or worsening infection-related manifestations, deterioration of organ function, escalation of antimicrobial therapy because of treatment failure, or death before achieving clinical response, were classified as non-responders. Thus, patients who died before achieving clinical response were not excluded solely because they had not reached a scheduled response-assessment time point. Only patients whose available medical records were insufficient to determine either clinical response or non-response were excluded during cohort construction.

To further address the time-varying nature of clinical response, we performed an additional Cox regression analysis in which clinical response was modeled as a time-dependent covariate. All patients entered the analysis in the non-response state at time zero. Patients who subsequently achieved clinical response remained in the non-response state until the first documented date of response and were classified as responders thereafter. Patients who never achieved clinical response remained in the non-response state throughout follow-up. Accordingly, patients who died before achieving a documented response experienced the mortality endpoint while still in the non-response state. No survival time before the actual occurrence of clinical response was attributed to the response state.

After accounting for the timing of clinical response, the absence of clinical response remained independently associated with a higher risk of both 28-day mortality (HR=5.632; 95%CI=3.698-8.578; p<0.001) and 90-day mortality (HR=2.966; 95%CI=2.205-3.990; p<0.001). These findings were consistent with the primary analysis and indicate that the association between clinical response and improved short-term survival was not solely attributable to immortal-time bias. We have clarified the response-assessment process and added the time-dependent sensitivity analysis to the Methods and Results sections of the revised manuscript.

2. In the Introduction section, the authors note that infection is a major precipitant of acute decompensation and ACLF in cirrhosis. Please consider adding a brief statement regarding infection susceptibility, mucosal barrier function and gut-liver axis-related mechanisms in patients with cirrhosis (eg. PMID: 40160254; PMID: 39524932).

Response: We thank the reviewer for this valuable suggestion. We agree that the increased susceptibility to infection in cirrhosis reflects not only systemic immune dysfunction but also impaired mucosal defenses and dysregulated communication along the gut-liver axis. We have therefore added a brief statement to the Introduction describing how disruption of goblet-cell and mucus barrier function, microbial dysbiosis, and perturbations of the gut-liver and oral-gut-liver axes may promote microbial translocation, systemic inflammation, and infectious complications.

3. The definition of clinical non-response appears to include modification or broadening of the initial empiric antibiotic regimen and the development of a new infectious focus during the same episode. Since the manuscript subsequently analyzes the associations between non-response, antibiotic escalation, secondary infection, and mortality, some degree of definitional overlap or reverse causality may exist. Please further clarify this issue.

Response: We thank the reviewer for identifying this important issue. We agree that the original wording could be interpreted as creating definitional overlap, particularly for antibiotic escalation, and could obscure the temporal relationship among response status, secondary infection, and mortality. We have therefore clarified the operational definitions and revised the interpretation of these analyses.

First, clinical response and non-response were determined primarily from serial changes in infection-related manifestations, organ dysfunction, and inflammatory markers. Modification or broadening of antibiotic therapy was not considered sufficient on its own to establish non-response. It was treated only as supportive evidence when the change was prompted by documented clinical or laboratory non-improvement. Antibiotic changes based solely on microbiological susceptibility results, drug toxicity, or antimicrobial-stewardship considerations did not determine response status.

Second, we distinguished progression or anatomical extension of the unresolved index infection from a secondary infection. A secondary infection was required to represent a clinically distinct new nosocomial episode arising after the index infection during the same hospitalization. Persistence, progression, or extension of the index infection was not counted as a secondary infection, and secondary infection was recorded separately rather than being used to classify response to the initial empiric regimen.

Third, because treatment escalation may be a consequence of perceived clinical failure, we removed the responder-versus-non-responder comparison of escalation from the Results and deleted causal interpretations suggesting that non-response independently led to broader antibiotic exposure. Any remaining information on escalation is presented only as a descriptive treatment characteristic.

Finally, the association with mortality was reassessed using clinical response as a time-dependent covariate, as described in our response to Comment 1. This analysis ensures that patients contribute person-time to the response state only after response has been documented. We have also acknowledged in the Limitations that, despite these clarifications, residual reverse causality cannot be completely excluded in a retrospective study.

Corresponding changes have been made in the Methods, Results, Discussion, and Limitations sections.

4. Given potential differences in microbiological testing, local resistance patterns, antibiotic strategies, ICU resources, and patient case mix across centers, please consider adjusting for center effects as a sensitivity analysis.

Response: We thank the reviewer for this important suggestion. We agree that differences in microbiological testing, local resistance patterns, antimicrobial strategies, ICU resources, and patient case mix may result in correlation among patients treated within the same center.

To account for potential within-center clustering, we performed sensitivity analyses in which the original multivariable logistic and Cox regression models were refitted using robust standard errors clustered by participating center. The same patient-level covariates as in the corresponding primary models were retained, without additional variable selection.

The center-clustered analyses yielded results consistent with the primary findings. The direction and magnitude of the principal associations with clinical non-response remained materially unchanged. In addition, lack of clinical response remained independently associated with both 28-day mortality (HR=3.984; 95%CI=2.526-6.283; p<0.001) and 90-day mortality (HR=3.083; 95%CI=2.242-4.242; p<0.001) after accounting for within-center correlation.

These findings suggest that the main associations were robust to clustering of patients within participating centers. However, we acknowledge that cluster-robust standard errors account for within-center correlation but cannot fully eliminate confounding from unmeasured institutional characteristics. The corresponding methods and results have been added to the revised manuscript.

5. In the Discussion section, the influence of systemic inflammation, infection profiles, local microbiology and inter-centre differences on the response to empiric antibiotic therapy were be discussed. The discussion could be modestly expanded to include the gut-liver axis, immune-inflammatory responses, organ-to-organ interactions, and regional/institutional variation, which may help explain the heterogeneity in treatment response and outcomes across patients, centres and geographic settings (eg. PMID: 39735421; PMID: 39243796).

Response: We thank the reviewer for this insightful suggestion. We agree that heterogeneity in the response to empiric antibiotic therapy is likely to reflect interactions between host pathophysiology, the infectious insult, and the regional or institutional healthcare context. We have therefore modestly expanded the Discussion to incorporate the gut-liver axis, immune-inflammatory dysregulation, organ-to-organ crosstalk, and health-system variation as potential explanations for the observed differences in treatment response and outcomes.

Specifically, we now discuss how intestinal barrier dysfunction and microbial dysbiosis may promote microbial translocation, while cirrhosis-associated immune dysfunction and excessive or dysregulated inflammatory responses may simultaneously impair pathogen clearance and amplify tissue injury. We also note that bidirectional interactions between the gut and liver, as well as crosstalk among the liver, kidneys, lungs, and systemic circulation, may propagate inflammation and organ failure and thereby influence the likelihood of response to initial antimicrobial therapy. Because these mechanisms were not directly measured in our cohort, they are presented as a biologically plausible interpretive framework rather than as causal findings of this study.

We have also expanded the discussion of regional and institutional heterogeneity. In addition to differences in patient case mix, cirrhosis etiology, infection acquisition, pathogen distribution, and antimicrobial resistance, variation in microbiological diagnostics, prescribing and antimicrobial-stewardship practices, source-control capacity, and access to critical care may affect treatment selection, timely adaptation of therapy, and clinical outcomes. These considerations further support locally informed empiric strategies and centre-specific surveillance.
