# Supplementary material for: Clinical efficacy of empiric antibiotic therapy in Chinese patients with cirrhosis and bacterial infections: A multicenter study
Source: PLoS One. 2026 Sep 15;21(9):e0357491. doi: 10.1371/journal.pone.0357491 (PMC13577579; doi:10.1371/journal.pone.0357491)
Supplement: Peer review history

# Peer review: Decision Letter 0

**DOI:** 10.1371/journal.pone.0357491.r001

**Authors:** Lanlan Chen

9 Jul 2026

Dear Dr. Shi,

Thank you for submitting your manuscript to PLOS ONE. After careful consideration, we feel that it has merit but does not fully meet PLOS ONE’s publication criteria as it currently stands. Therefore, we invite you to submit a revised version of the manuscript that addresses the points raised during the review process.

Please submit your revised manuscript by Sep 07 2026 11:59PM. If you will need more time than this to complete your revisions, please reply to this message or contact the journal office at plosone@plos.org. When you're ready to submit your revision, log on to https://www.editorialmanager.com/pone/ and select the 'Submissions Needing Revision' folder to locate your manuscript file.

- A letter that responds to each point raised by the academic editor and reviewer(s). You should upload this letter as a separate file labeled 'Response to Reviewers'.
- A marked-up copy of your manuscript that highlights changes made to the original version. You should upload this as a separate file labeled 'Revised Manuscript with Track Changes'.
- An unmarked version of your revised paper without tracked changes. You should upload this as a separate file labeled 'Manuscript'.

If you would like to make changes to your financial disclosure, please include your updated statement in your cover letter. Guidelines for resubmitting your figure files are available below the reviewer comments at the end of this letter.

If applicable, we recommend that you deposit your laboratory protocols in protocols.io to enhance the reproducibility of your results. Protocols.io assigns your protocol its own identifier (DOI) so that it can be cited independently in the future. For instructions see: https://journals.plos.org/plosone/s/submission-guidelines#loc-laboratory-protocols. Additionally, PLOS ONE offers an option for publishing peer-reviewed Lab Protocol articles, which describe protocols hosted on protocols.io. Read more information on sharing protocols at https://plos.org/protocols?utm_medium=editorial-email&utm_source=authorletters&utm_campaign=protocols.

As the corresponding author, your ORCID iD is verified in the submission system and will appear in the published article. PLOS supports the use of ORCID, and we encourage all coauthors to register for an ORCID iD and use it as well. Please encourage your coauthors to verify their ORCID iD within the submission system before final acceptance, as unverified ORCID iDs will not appear in the published article. *Only*  the individual author can complete the verification step; PLOS staff *cannot*  verify ORCID iDs on behalf of authors.

We look forward to receiving your revised manuscript.

Kind regards,

Lanlan Chen

Academic Editor

PLOS One

Journal Requirements:

When submitting your revision, we need you to address these additional requirements.

1. Please ensure that your manuscript meets PLOS ONE's style requirements, including those for file naming. The PLOS ONE style templates can be found at

https://journals.plos.org/plosone/s/file?id=wjVg/PLOSOne_formatting_sample_main_body.pdf and

https://journals.plos.org/plosone/s/file?id=ba62/PLOSOne_formatting_sample_title_authors_affiliations.pdf

2. Thank you for stating the following in the Acknowledgments Section of your manuscript:

“This work was supported by the Prevention and Control of Emerging and Major Infectious Diseases-National Science and Technology Major Project (2025ZD01906200), and the National Natural Science Foundation of China (82570717).”

We note that you have provided funding information that is not currently declared in your Funding Statement. However, funding information should not appear in the Acknowledgments section or other areas of your manuscript. We will only publish funding information present in the Funding Statement section of the online submission form.

Please remove any funding-related text from the manuscript and let us know how you would like to update your Funding Statement. Currently, your Funding Statement reads as follows:

“This work was supported by the Prevention and Control of Emerging and Major Infectious Diseases-National Science and Technology Major Project (2025ZD01906200), and the National Natural Science Foundation of China (82570717).”

Please include your amended statements within your cover letter; we will change the online submission form on your behalf.

3. We note that the grant information you provided in the ‘Funding Information’ and ‘Financial Disclosure’ sections do not match.

When you resubmit, please ensure that you provide the correct grant numbers for the awards you received for your study in the ‘Funding Information’ section.

4. Thank you for stating the following financial disclosure:

“This work was supported by the Prevention and Control of Emerging and Major Infectious Diseases-National Science and Technology Major Project (2025ZD01906200), and the National Natural Science Foundation of China (82570717).”

Please state what role the funders took in the study.  If the funders had no role, please state: "The funders had no role in study design, data collection and analysis, decision to publish, or preparation of the manuscript."

If this statement is not correct you must amend it as needed.

Please include this amended Role of Funder statement in your cover letter; we will change the online submission form on your behalf.

5. In the online submission form you indicate that your data is not available for proprietary reasons and have provided a contact point for accessing this data. Please note that your current contact point is a co-author on this manuscript. According to our Data Policy, the contact point must not be an author on the manuscript and must be an institutional contact, ideally not an individual. Please revise your data statement to a non-author institutional point of contact, such as a data access or ethics committee, and send this to us via return email. Please also include contact information for the third party organization, and please include the full citation of where the data can be found.

6. PLOS requires an ORCID iD for the corresponding author in Editorial Manager on papers submitted after December 6th, 2016. Please ensure that you have an ORCID iD and that it is validated in Editorial Manager. To do this, go to ‘Update my Information’ (in the upper left-hand corner of the main menu), and click on the Fetch/Validate link next to the ORCID field. This will take you to the ORCID site and allow you to create a new iD or authenticate a pre-existing iD in Editorial Manager.

7. Your ethics statement should only appear in the Methods section of your manuscript. If your ethics statement is written in any section besides the Methods, please delete it from any other section.

8. Please include captions for your Supporting Information files at the end of your manuscript, and update any in-text citations to match accordingly. Please see our Supporting Information guidelines for more information: http://journals.plos.org/plosone/s/supporting-information.

9. If the reviewer comments include a recommendation to cite specific previously published works, please review and evaluate these publications to determine whether they are relevant and should be cited. There is no requirement to cite these works unless the editor has indicated otherwise.

[Note: HTML markup is below. Please do not edit.]

Reviewers' comments:

Reviewer's Responses to Questions

**Comments to the Author**

1. Is the manuscript technically sound, and do the data support the conclusions?

Reviewer #1: Yes

**********

2. Has the statistical analysis been performed appropriately and rigorously?

Reviewer #1: Yes

**********

3. Have the authors made all data underlying the findings in their manuscript fully available?

The PLOS Data policy

Reviewer #1: Yes

**********

4. Is the manuscript presented in an intelligible fashion and written in standard English?

Reviewer #1: Yes

**********

Reviewer #1: This multicenter retrospective study evaluates the clinical efficacy of empiric antibiotic therapy in Chinese patients with cirrhosis and bacterial or fungal infections. Based on a large cohort from 24 tertiary centers, the authors describe the rate of early clinical response, identify clinical and inflammatory predictors of non-response, and further examine the associations of treatment response and secondary infection with short-term mortality. Overall, the study addresses a clinically relevant question and provides useful real-world data for risk-adapted antibiotic management in cirrhosis. However, several issues might be further resolved.

Clinical response is a central exposure in this study and is strongly associated with 28-day and 90-day mortality. Since the median time to clinical response was 5 days, it would be helpful to clarify the exact time window used to define response and how early deaths before response assessment were handled. Please consider performing a sensitivity analysis treating clinical response as a time-dependent variable in the Cox model.

In the Introduction section, the authors note that infection is a major precipitant of acute decompensation and ACLF in cirrhosis. Please consider adding a brief statement regarding infection susceptibility, mucosal barrier function and gut-liver axis-related mechanisms in patients with cirrhosis (eg. PMID: 40160254; PMID: 39524932).

The definition of clinical non-response appears to include modification or broadening of the initial empiric antibiotic regimen and the development of a new infectious focus during the same episode. Since the manuscript subsequently analyzes the associations between non-response, antibiotic escalation, secondary infection, and mortality, some degree of definitional overlap or reverse causality may exist. Please further clarify this issue.

Given potential differences in microbiological testing, local resistance patterns, antibiotic strategies, ICU resources, and patient case mix across centers, please consider adjusting for center effects as a sensitivity analysis.

In the Discussion section, the influence of systemic inflammation, infection profiles, local microbiology and inter-centre differences on the response to empiric antibiotic therapy were be discussed. The discussion could be modestly expanded to include the gut-liver axis, immune-inflammatory responses, organ-to-organ interactions, and regional/institutional variation, which may help explain the heterogeneity in treatment response and outcomes across patients, centres and geographic settings (eg. PMID: 39735421; PMID: 39243796).

**********

what does this mean?). If published, this will include your full peer review and any attached files.

If you choose “no”, your identity will remain anonymous but your review may still be made public.

**Do you want your identity to be public for this peer review?**  For information about this choice, including consent withdrawal, please see our Privacy Policy

Reviewer #1: No

**********

[NOTE: If reviewer comments were submitted as an attachment file, they will be attached to this email and accessible via the submission site. Please log into your account, locate the manuscript record, and check for the action link "View Attachments". If this link does not appear, there are no attachment files.]

To ensure your figures meet our technical requirements, please review our figure guidelines: https://journals.plos.org/plosone/s/figures

You may also use PLOS’s free figure tool, NAAS, to help you prepare publication quality figures: https://journals.plos.org/plosone/s/figures#loc-tools-for-figure-preparation.

NAAS will assess whether your figures meet our technical requirements by comparing each figure against our figure specifications.

# Author comment: Author response to Decision Letter 1

**DOI:** 10.1371/journal.pone.0357491.r002

14 Aug 2026

Revision for “Clinical efficacy of empiric antibiotic therapy in Chinese patients with cirrhosis and bacterial infections: a multicenter study” (Manuscript ID: PONE-D-26-28291)

Dear Editor,

We appreciate the opportunity to respond to the reviewers’ comments and to improve our manuscript entitled, “Clinical efficacy of empiric antibiotic therapy in Chinese patients with cirrhosis and bacterial infections: a multicenter study.” We have addressed each comment by all reviewers as below, with changes underlined and highlighted by yellow color. We hope that the resubmitted manuscript is acceptable for publication.

Funding Statement：

This work was supported by the National Major Science and Technology Projects of China (2025ZD01906200), and the National Natural Science Foundation of China (82570717).

Role of Funder statement：

The funders had no role in study design, data collection and analysis, decision to publish, or preparation of the manuscript.

Sincerely,

Prof. Xia Yu, MD

Email: zjuyuxia@zju.edu.cn

State Key Laboratory for Diagnosis and Treatment of Infectious Diseases,

National Clinical Research Center for Infectious Diseases,

National Medical Center for Infectious Diseases,

Collaborative Innovation Center for Diagnosis and Treatment of Infectious Diseases, The First Affiliated Hospital, College of Medicine, Zhejiang University

RESPONSES OF THE AUTHORS TO REVIEWER’S COMMENTS:

REVIEWER'S COMMENTS TO AUTHOR:

Reviewer #1:

This multicenter retrospective study evaluates the clinical efficacy of empiric antibiotic therapy in Chinese patients with cirrhosis and bacterial or fungal infections. Based on a large cohort from 24 tertiary centers, the authors describe the rate of early clinical response, identify clinical and inflammatory predictors of non-response, and further examine the associations of treatment response and secondary infection with short-term mortality. Overall, the study addresses a clinically relevant question and provides useful real-world data for risk-adapted antibiotic management in cirrhosis. However, several issues might be further resolved.

1.Clinical response is a central exposure in this study and is strongly associated with 28-day and 90-day mortality. Since the median time to clinical response was 5 days, it would be helpful to clarify the exact time window used to define response and how early deaths before response assessment were handled. Please consider performing a sensitivity analysis treating clinical response as a time-dependent variable in the Cox model.

Response: Thank you for this important comment. Clinical response was not assessed at a single prespecified time point. Instead, it was evaluated longitudinally from the initiation of first-line empiric antibiotic therapy, which was defined as time zero, until hospital discharge or in-hospital death. During hospitalization, patients underwent repeated clinical assessment and laboratory testing according to routine clinical practice, generally at intervals of approximately 1-2 days. The date of clinical response was defined as the first date on which the patient met the prespecified clinical and laboratory response criteria.

In the primary analysis, patients were classified as responders if they demonstrated documented improvement in infection- or inflammation-related manifestations, inflammatory markers, and infection-related organ dysfunction during the index hospitalization. Patients who did not achieve a documented response before hospital discharge, or who experienced persistent or worsening infection-related manifestations, deterioration of organ function, escalation of antimicrobial therapy because of treatment failure, or death before achieving clinical response, were classified as non-responders. Thus, patients who died before achieving clinical response were not excluded solely because they had not reached a scheduled response-assessment time point. Only patients whose available medical records were insufficient to determine either clinical response or non-response were excluded during cohort construction.

To further address the time-varying nature of clinical response, we performed an additional Cox regression analysis in which clinical response was modeled as a time-dependent covariate. All patients entered the analysis in the non-response state at time zero. Patients who subsequently achieved clinical response remained in the non-response state until the first documented date of response and were classified as responders thereafter. Patients who never achieved clinical response remained in the non-response state throughout follow-up. Accordingly, patients who died before achieving a documented response experienced the mortality endpoint while still in the non-response state. No survival time before the actual occurrence of clinical response was attributed to the response state.

After accounting for the timing of clinical response, the absence of clinical response remained independently associated with a higher risk of both 28-day mortality (HR=5.632; 95%CI=3.698-8.578; p<0.001) and 90-day mortality (HR=2.966; 95%CI=2.205-3.990; p<0.001). These findings were consistent with the primary analysis and indicate that the association between clinical response and improved short-term survival was not solely attributable to immortal-time bias. We have clarified the response-assessment process and added the time-dependent sensitivity analysis to the Methods and Results sections of the revised manuscript.

2.In the Introduction section, the authors note that infection is a major precipitant of acute decompensation and ACLF in cirrhosis. Please consider adding a brief statement regarding infection susceptibility, mucosal barrier function and gut-liver axis-related mechanisms in patients with cirrhosis (eg. PMID: 40160254; PMID: 39524932).

Response: We thank the reviewer for this valuable suggestion. We agree that the increased susceptibility to infection in cirrhosis reflects not only systemic immune dysfunction but also impaired mucosal defenses and dysregulated communication along the gut-liver axis. We have therefore added a brief statement to the Introduction describing how disruption of goblet-cell and mucus barrier function, microbial dysbiosis, and perturbations of the gut-liver and oral-gut-liver axes may promote microbial translocation, systemic inflammation, and infectious complications.

3.The definition of clinical non-response appears to include modification or broadening of the initial empiric antibiotic regimen and the development of a new infectious focus during the same episode. Since the manuscript subsequently analyzes the associations between non-response, antibiotic escalation, secondary infection, and mortality, some degree of definitional overlap or reverse causality may exist. Please further clarify this issue.

Response: We thank the reviewer for identifying this important issue. We agree that the original wording could be interpreted as creating definitional overlap, particularly for antibiotic escalation, and could obscure the temporal relationship among response status, secondary infection, and mortality. We have therefore clarified the operational definitions and revised the interpretation of these analyses.

First, clinical response and non-response were determined primarily from serial changes in infection-related manifestations, organ dysfunction, and inflammatory markers. Modification or broadening of antibiotic therapy was not considered sufficient on its own to establish non-response. It was treated only as supportive evidence when the change was prompted by documented clinical or laboratory non-improvement. Antibiotic changes based solely on microbiological susceptibility results, drug toxicity, or antimicrobial-stewardship considerations did not determine response status.

Second, we distinguished progression or anatomical extension of the unresolved index infection from a secondary infection. A secondary infection was required to represent a clinically distinct new nosocomial episode arising after the index infection during the same hospitalization. Persistence, progression, or extension of the index infection was not counted as a secondary infection, and secondary infection was recorded separately rather than being used to classify response to the initial empiric regimen.

Third, because treatment escalation may be a consequence of perceived clinical failure, we removed the responder-versus-non-responder comparison of escalation from the Results and deleted causal interpretations suggesting that non-response independently led to broader antibiotic exposure. Any remaining information on escalation is presented only as a descriptive treatment characteristic.

Finally, the association with mortality was reassessed using clinical response as a time-dependent covariate, as described in our response to Comment 1. This analysis ensures that patients contribute person-time to the response state only after response has been documented. We have also acknowledged in the Limitations that, despite these clarifications, residual reverse causality cannot be completely excluded in a retrospective study.

Corresponding changes have been made in the Methods, Results, Discussion, and Limitations sections.

4.Given potential differences in microbiological testing, local resistance patterns, antibiotic strategies, ICU resources, and patient case mix across centers, please consider adjusting for center effects as a sensitivity analysis.

Response: We thank the reviewer for this important suggestion. We agree that differences in microbiological testing, local resistance patterns, antimicrobial strategies, ICU resources, and patient case mix may result in correlation among patients treated within the same center.

To account for potential within-center clustering, we performed sensitivity analyses in which the original multivariable logistic and Cox regression models were refitted using robust standard errors clustered by participating center. The same patient-level covariates as in the corresponding primary models were retained, without additional variable selection.

The center-clustered analyses yielded results consistent with the primary findings. The direction and magnitude of the principal associations with clinical non-response remained materially unchanged. In addition, lack of clinical response remained independently associated with both 28-day mortality (HR=3.984; 95%CI=2.526-6.283; p<0.001) and 90-day mortality (HR=3.083; 95%CI=2.242-4.242; p<0.001) after accounting for within-center correlation.

These findings suggest that the main associations were robust to clustering of patients within participating centers. However, we acknowledge that cluster-robust standard errors account for within-center correlation but cannot fully eliminate confounding from unmeasured institutional characteristics. The corresponding methods and results have been added to the revised manuscript.

5.In the Discussion section, the influence of systemic inflammation, infection profiles, local microbiology and inter-centre differences on the response to empiric antibiotic therapy were be discussed. The discussion could be modestly expanded to include the gut-liver axis, immune-inflammatory responses, organ-to-organ interactions, and regional/institutional variation, which may help explain the heterogeneity in treatment response and outcomes across patients, centres and geographic settings (eg. PMID: 39735421; PMID: 39243796).

Response: We thank the reviewer for this insightful suggestion. We agree that heterogeneity in the response to empiric antibiotic therapy is likely to reflect interactions between host pathophysiology, the infectious insult, and the regional or institutional healthcare context. We have therefore modestly expanded the Discussion to incorporate the gut-liver axis, immune-inflammatory dysregulation, organ-to-organ crosstalk, and health-system variation as potential explanations for the observed differences in treatment response and outcomes.

Specifically, we now discuss how intestinal barrier dysfunction and microbial dysbiosis may promote microbial translocation, while cirrhosis-associated immune dysfunction and excessive or dysregulated inflammatory responses may simultaneously impair pathogen clearance and amplify tissue injury. We also note that bidirectional interactions between the gut and liver, as well as crosstalk among the liver, kidneys, lungs, and systemic circulation, may propagate inflammation and organ failure and thereby influence the likelihood of response to initial antimicrobial therapy. Because these mechanisms were not directly measured in our cohort, they are presented as a biologically plausible interpretive framework rather than as causal findings of this study.

We have also expanded the discussion of regional and institutional heterogeneity. In addition to differences in patient case mix, cirrhosis etiology, infection acquisition, pathogen distribution, and antimicrobial resistance, variation in microbiological diagnostics, prescribing and antimicrobial-stewardship practices, source-control capacity, and access to critical care may affect treatment selection, timely adaptation of therapy, and clinical outcomes. These considerations further support locally informed empiric strategies and centre-specific surveillance.

Attachment Submitted filename: Response to Reviewers.docx

# Peer review: Decision Letter 1

**DOI:** 10.1371/journal.pone.0357491.r003

**Authors:** Lanlan Chen

18 Aug 2026

Clinical efficacy of empiric antibiotic therapy in Chinese patients with cirrhosis and bacterial infections: a multicenter study

PONE-D-26-28291R1

Dear Dr. Shi,

We’re pleased to inform you that your manuscript has been judged scientifically suitable for publication and will be formally accepted for publication once it meets all outstanding technical requirements.

Within one week, you’ll receive an e-mail detailing the required amendments. When these have been addressed, you’ll receive a formal acceptance letter and your manuscript will be scheduled for publication.

An invoice will be generated when your article is formally accepted. Please note, if your institution has a publishing partnership with PLOS and your article meets the relevant criteria, all or part of your publication costs will be covered. Please make sure your user information is up-to-date by logging into Editorial Manager at Editorial Manager® and clicking the ‘Update My Information' link at the top of the page. For questions related to billing, please contact billing support.

If your institution or institutions have a press office, please notify them about your upcoming paper to help maximize its impact. If they’ll be preparing press materials, please inform our press team as soon as possible -- no later than 48 hours after receiving the formal acceptance. Your manuscript will remain under strict press embargo until 2 pm Eastern Time on the date of publication. For more information, please contact onepress@plos.org.

Kind regards,

Lanlan Chen

Academic Editor

PLOS One

Additional Editor Comments (optional):

Reviewers' comments:

Reviewer's Responses to Questions

**Comments to the Author**

Reviewer #1: All comments have been addressed

**********

2. Is the manuscript technically sound, and do the data support the conclusions?

Reviewer #1: Yes

**********

3. Has the statistical analysis been performed appropriately and rigorously?

Reviewer #1: Yes

**********

4. Have the authors made all data underlying the findings in their manuscript fully available?

The PLOS Data policy

Reviewer #1: Yes

**********

5. Is the manuscript presented in an intelligible fashion and written in standard English?

Reviewer #1: Yes

**********

Reviewer #1: I have finished my evaluation of the revised manuscript. The authors have responded thoroughly to every comment, and the corresponding revisions are appropriate and well‑prepared. No further modifications are required from my perspective, and I do not have any more reviewer comments.

**********

what does this mean?). If published, this will include your full peer review and any attached files.

If you choose “no”, your identity will remain anonymous but your review may still be made public.

**Do you want your identity to be public for this peer review?**  For information about this choice, including consent withdrawal, please see our Privacy Policy

Reviewer #1: No

**********

# Editor report: Acceptance letter

**DOI:** 10.1371/journal.pone.0357491.r004

**Authors:** Lanlan Chen

PONE-D-26-28291R1

PLOS One

Dear Dr. Shi,

I'm pleased to inform you that your manuscript has been deemed suitable for publication in PLOS One. Congratulations! Your manuscript is now being handed over to our production team.

At this stage, our production department will prepare your paper for publication. This includes ensuring the following:

* All references, tables, and figures are properly cited

* All relevant supporting information is included in the manuscript submission,

* There are no issues that prevent the paper from being properly typeset

You will receive further instructions from the production team, including instructions on how to review your proof when it is ready. Please keep in mind that we are working through a large volume of accepted articles, so please give us a few days to review your paper and let you know the next and final steps.

Lastly, if your institution or institutions have a press office, please let them know about your upcoming paper now to help maximize its impact. If they'll be preparing press materials, please inform our press team within the next 48 hours. Your manuscript will remain under strict press embargo until 2 pm Eastern Time on the date of publication. For more information, please contact onepress@plos.org.

You will receive an invoice from PLOS for your publication fee after your manuscript has reached the completed accept phase. If you receive an email requesting payment before acceptance or for any other service, this may be a phishing scheme. Learn how to identify phishing emails and protect your accounts at https://explore.plos.org/phishing.

If we can help with anything else, please email us at customercare@plos.org.

Thank you for submitting your work to PLOS One and supporting open access.

Kind regards,

PLOS One Editorial Office Staff

on behalf of

M.D Lanlan Chen

Academic Editor

PLOS One
